# Microbiome diversity declines while distinct expansions of Th17, iNKT, and dendritic cell subpopulations emerge after anastomosis surgery

**DOI:** 10.1186/s13099-021-00447-z

**Published:** 2021-08-10

**Authors:** Emilie E. Vomhof-DeKrey, Allie Stover, Marc D. Basson

**Affiliations:** 1 Department of Surgery, University of North Dakota School of Medicine and the Health Sciences, 1301 North Columbia Road, Stop 9037, Grand Forks, ND 58202 USA; 2 Department of Biomedical Sciences, University of North Dakota School of Medicine and the Health Sciences, 1301 North Columbia Road, Stop 9037, Grand Forks, ND 58202 USA; 3 Department of Pathology, University of North Dakota School of Medicine and the Health Sciences, 1301 North Columbia Road, Stop 9037, Grand Forks, ND 58202 USA

**Keywords:** Immune-microbiota crosstalk, Mucosal healing, Toll-like receptors, CD11b^hi^ CD103^mid^

## Abstract

**Background:**

Anastomotic failure causes morbidity and mortality even in technically correct anastomoses. Initial leaks must be prevented by mucosal reapproximation across the anastomosis. Healing is a concerted effort between intestinal epithelial cells (IECs), immune cells, and commensal bacteria. IEC TLR4 activation and signaling is required for mucosal healing, leading to inflammatory factor release that recruits immune cells to limit bacteria invasion. TLR4 absence leads to mucosal damage from loss in epithelial proliferation, attenuated inflammatory response, and bacteria translocation. We hypothesize after anastomosis, an imbalance in microbiota will occur due to a decrease in TLR4 expression and will lead to changes in the immune milieu.

**Results:**

We isolated fecal content and small intestinal leukocytes from murine, Roux-en-Y and end-to-end anastomoses, to identify microbiome changes and subsequent alterations in the regulatory and pro-inflammatory immune cells 3 days post-operative. TLR4^+^ IECs were impaired after anastomosis. Microbiome diversity was reduced, with *Firmicutes, Bacteroidetes*, and *Saccharibacteria* decreased and *Proteobacteria* increased. A distinct TCRβ^hi^ CD4^+^ T cells subset after anastomosis was 10–20-fold greater than in control mice. 84% were Th17 IL-17A/F^+^ IL-22^+^ and/or TNFα^+^. iNKT cells were increased and TCRβ^hi^. 75% were iNKT IL-10^+^ and 13% iNKTh17 IL-22^+^. Additionally, Treg IL-10^+^ and IL-22^+^ cells were increased. A novel dendritic cell subset was identified in anastomotic regions that was CD11b^hi^ CD103^mid^ and was 93% IL-10^+^.

**Conclusions:**

This anastomotic study demonstrated a decrease in IEC TLR4 expression and microbiome diversity which then coincided with increased expansion of regulatory and pro-inflammatory immune cells and cytokines. Defining the anastomotic mucosal environment could help inform innovative therapeutics to target excessive pro-inflammatory invasion and microbiome imbalance.

**Supplementary Information:**

The online version contains supplementary material available at 10.1186/s13099-021-00447-z.

## Background

Anastomotic failure is one of the most serious complications of intestinal surgery [24]. An anastomotic leak can lead to high morbidity, increased mortality, and considerable added hospital costs [18]. Even with extensive research and surgical technique improvements, small intestinal and colorectal anastomosis leakage can occur in 0.05–30% of operations [18, 24, 30, 31, 43]. Anastomotic leakage can vary in its onset of occurrence. An early leak within the first to second postoperative days occurs most often because of technical reasons, whereas a latent leak occurring by the end of the first postoperative week is most often attributed to a failure in the normal healing mechanisms [43].

Proper intestinal healing requires a coordinated crosstalk between intestinal epithelial cells (IECs), immune cells, and microbiome. The multifaceted toll-like receptor (TLR) signaling pathways serve as an interface for concerted crosstalk between the intestinal epithelial barrier, microbiota, and immune system [14]. The TLR signaling pathways help to maintain a constant homeostatic regulation of microbial load and the immune response. There are 10 total TLRs, and they are expressed on macrophages, dendritic cells (DCs), T lymphocytes, and IECs [14]. TLR4 is expressed in the apical region of the terminal ileum of mice [1]. TLR4 signaling is required for induction of mucosal healing pathways, the defense against gram-negative bacteria, and maintaining the tolerance to commensal bacteria. During mucosal injury, TLR4 activation on IECs leads to the release of inflammatory cytokines (i.e., IL-8) and chemokines that recruit innate and adaptive immune cells to limit bacterial invasion. TLR4 absence can lead to severe mucosal damage from a loss in epithelial proliferation, impaired inflammatory response, and bacterial translocation. However, TLR4 signaling needs to be tightly regulated as prolonged activation can result in detrimental inflammation that inhibits mucosal repair through decreased enterocyte proliferation and migration [14].

Many immune cell types function alongside gut microbiota to promote mucosal healing in the steady state and during injury. These include CD103^+^ conventional DCs (cDCs), Th17 cells, T regulatory (Treg) cells, iNKT, and iNKTh17 cells [24]. Typically, the gut microbiota induces differentiation of these immune cells. Treg and iNKT cells will undergo differentiation and secrete the anti-inflammatory cytokine, IL-10, through interactions with *Clostridia* [25]. The promotion of CD4^+^ T cells to Th17 cells can be induced by segmented filamentous bacteria (SFB) and Th17 and iNKTh17 cells will produce IL-17a, IL-17f, and IL-22, which is a key cytokine that acts on IL-22R only on epithelial cells in order to promote healing [25]. However, there is a dichotomy to this system, if SFB colonization it too expressive, systemic Th17 activation will result in increased inflammation and mucosal injury [25, 56]. Therefore, the IL-10 signaling of CD103^+^ DCs and Tregs are key to helping to regulate the Th17 activation [40]. Transforming growth factor-β drives the formation of Th17 cells from naïve T cells [34]. Th17 cells are known to produce cytokines IL-17 and IL-22 which have several roles in the gastrointestinal tract, such as cell proliferation, tissue regeneration, pathogen defense, and intestinal barrier maintenance and protection [22, 34]. IL-22 promotes production of innate antimicrobial molecules, like defensins, Reg family molecules, and S100 proteins by IECs [34]. Additionally, the intestinal epithelial barrier is maintained via a cross talk between IL-22, innate lymphoid cells type 3, and microbiota.

iNKT cells are subgroup of unconventional, CD1d-restricted T cells that have TCRs and are able to recognize endogenous and exogenous lipids, such as αGalCer, which are presented by the surface molecule CD1d in mice (CD1 in humans) [44]. After iNKT cell activation, copious amounts of cytokines such as IL-4, IL-10, and IL-22 are secreted to regulate the downstream activation of DCs, NK cells, B cells, or conventional T cells. Recently, there has been growing evidence that iNKT cells play a central role in governing the bidirectional interactions of the host cells and the commensal microbiota, which is key to intestinal homeostasis and preventing inflammation [13]. The modulation of the mucosal immunity and regulation of bacterial colonization is through iNKT cells recognizing the presentation of commensal-derived lipids by CD1d that is expressed on B cells, DCs, macrophages, IECs, and innate lymphoid cells [4, 13, 32, 33, 42, 44, 47, 53]. Despite all of the research that has been done to demonstrate intestinal iNKT cell function in intestinal homeostasis, there is still much that remains to be determined as to how iNKT cells populations adjust after intestinal surgery and how these changes correlate to changes in the intestinal commensal bacteria.

CD103^+^ DCs are a heterogeneous population and can be categorized into two subsets based on their expression of CD11b. Both populations, CD103^+^ CD11b^+^ and CD103^+^ CD11b^−^ are found in the small intestinal lamina propria and intestinal lymph, but CD103^+^ CD11b^+^ make up the majority of the population found in the lamina propria [8, 15, 20]. The CD103^+^ DCs are tolerogenic and are able to induce the differentiation of naïve T cells to FoxP3^+^ Treg cells, while CD103^−^ DCs are not toleragenic [8, 11, 49]. The intestinal CD103^+^ DC induction of Treg differentiation is dependent on retinoic acid and active transforming growth factor (TGF) b [8, 11, 49].

In this study, we performed two types of anastomosis surgeries on C57BL/6 wildtype mice, Roux-en-Y and end-to-end anastomoses. The focus of this manuscript is to discern the correlative relationship of the microbiota and immune system early after an anastomosis operation. We chose to evaluate these parameters at 3 days post-operative as this is the time when a latent leak could become clinically apparent [43]. Dissecting the regulatory mechanisms of mucosal healing could impact therapeutic treatments given after an anastomotic operation.

## Methods

### Mice and surgery

**Fig. 1:**
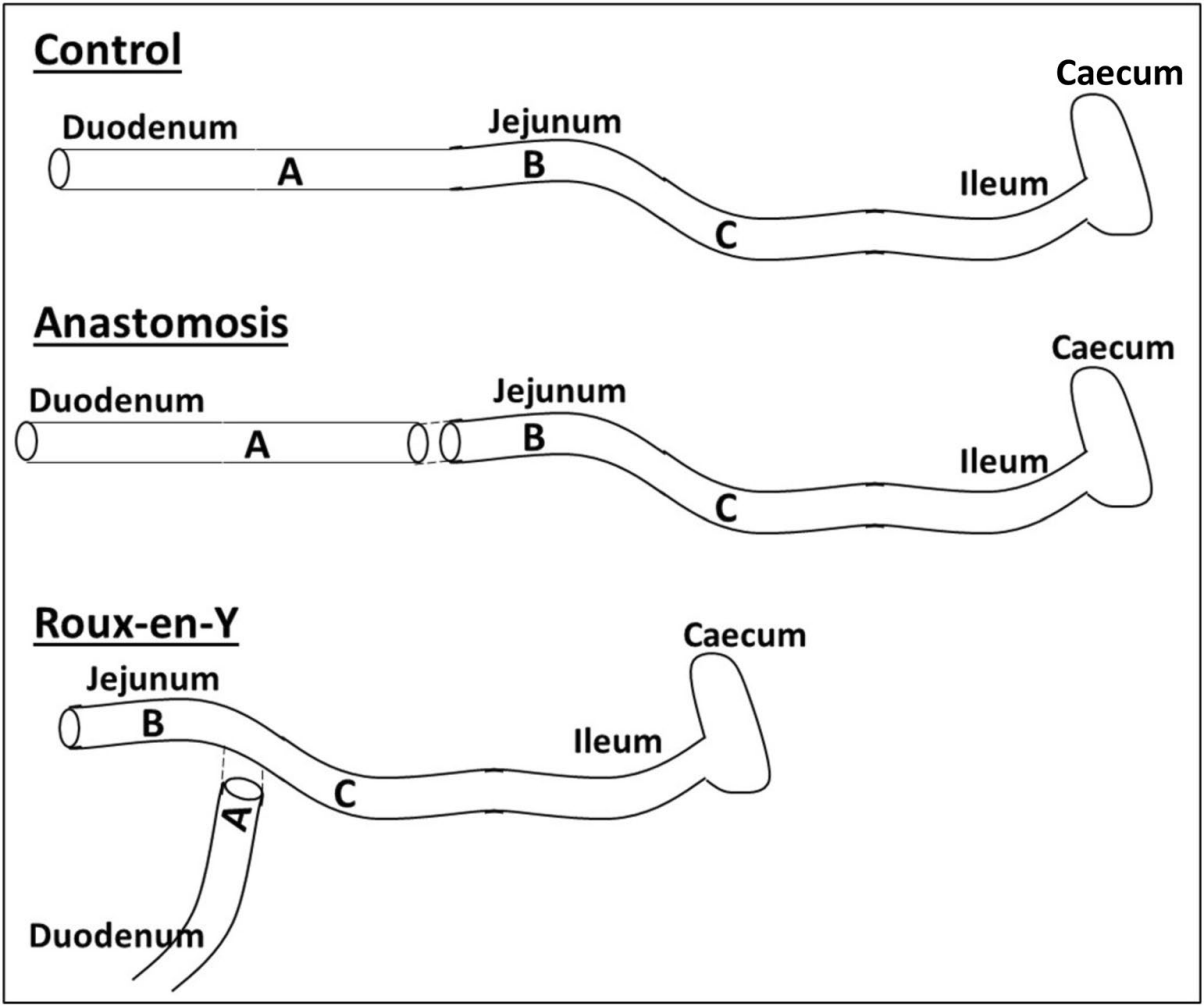
Surgical pictograms with intestinal segments labelled A-distal duodenum, B-jejunum/limb, and C-proximal ileum

Female C57Bl/6J mice, 7–14 weeks of age, were acquired from Jackson Laboratories (Bar Harbor, Maine) and bred at UND. These studies were performed according to protocol #1708-1 that was approved by the University of North Dakota Institutional Animal Care and Use Committee. Liquid diet (Vivonex Pediatric) was given to mice undergoing surgery 72 h prior to and the 3 days after surgery. For pain relief, 1–2 mg/kg of buprenorphine-sustained release was injected subcutaneously. Mice were anesthetized with 1–2% isoflurane and a laparotomy was performed. An end-to-end or Roux-en-Y anastomosis was made starting 5 cm from the pyloric orifice of the stomach using 8–0 Nylon suture (Unify, ADSurgical, black thread, taper point, 3/8 6.4 mm curved needle) (Fig. 1) [52]. The Roux-en-Y limb was approximately 2–3 cm long. A 5–0 Polysorb Braided Absorbable suture (Coviden, 18′′, 45 cm, undyed, P-12 cutting 3/8 19 mm needle, curved) was used to suture the body cavity and a 3–0 Perma-hand silk thread (Ethicon, black braided, 30′′ KS 60 mm straight, reverse cutting needle) was used to suture the skin. Mice were euthanized 3 days after surgery, intestinal fecal content and intestinal regions were harvested from the duodenum (A), jejunum (B), and ileum (C) as laid out in Fig. 1.

### Intestinal epithelial cell isolation

We isolated the intestinal cells using a protocol similar to Goodyear et al. [16]. Intestinal regions were cut open longitudinally and cut into 2 cm pieces and placed in a 50 ml tube with 10 ml complete RPMI (cRPMI: 500 ml RPMI, 10% FBS, 2 mM L-glutamine, 1 mM sodium pyruvate, 1  ×  non-essential amino acids, and PSEPx  =  100 units/ml penicillin, 100 µg/ml streptomycin, 25 µg/ml enrofloxacin, 100 units/ml polymyxin B (RPMI & FBS, Genesee Scientific, Cajon, CA; all other cRMPI components from Millipore Sigma, St. Louis, MO). Mucus was removed by washing tissue with Solution 1 (5 mM DTT in HBSS (Millipore Sigma)  +  PSEPx  +  2% FBS) at 37 °C with 20 ml/g tissue for 20 min/g tissue with 200 rpm shaking. This wash was discarded and then the epithelial layer was collected with 3 washes at 37 °C of Solution 2 (5 mM EDTA in HBSS  +  PSEPx  +  2% FBS) using 15 ml/g of tissue for 15 min/g of tissue and 200 rpm shaking. Each wash was collected in a 50 ml tube and stored on ice to combine with the lamina propria cells after the next step. The tissue was then washed with 10 ml Solution 3 (HBSS  +  PSEPx  +  10 mM HEPES) for 10 min with 200 rpm shaking in order to remove the FBS and EDTA, this solution was discarded after this wash. To collect a lamina propria single cell suspension from the remaining tissue, 4 ml of 1  ×  Liberase TM (0.2 Wunsch unit/ml, Millipore Sigma) + 200 K/ml DNase I (Millipore Sigma) in Solution 3 was added and sample was incubated at 37 °C for 15 min, with 200 rpm shaking. Following digestion, 2 ml of cRPMI was added and then samples were triturated 3 times through an 18-gauge needle and then samples were filtered through a 70 µm sieve. The filtrate was combined with the epithelial cells from above and centrifuged at 300×*g* for 5 min, 4 °C. Cells were then stained for flow cytometry analysis.

### Flow cytometry

Epithelial and lamina propria lymphocyte cells were stained with the viability Zombie Aqua dye from Biolegend (San Diego, CA) per manufacturer’s protocol. Then cells were pretreated with TruStain fcX (Biolegend) in order to block non-specific binding of antibodies to Fc receptors. Mouse CD1d-αGalCer tetramers (PBS57; NIH Tetramer Core Facility) conjugated to Brilliant Violet 421 (BV421) were utilized to identify iNKT cells (TCRβ^+^ CD1dtet^+^). All of the following mouse antibodies were purchased from Biolegend except if noted differently. The following mouse antibodies were used to stain for Tregs, Th17, and iNKTh17 cells: anti-TCRβ (H57-597), anti-CD4 (GK1.5). The following mouse antibodies were used to stain for DCs: anti-CD45 (30-F11), anti-CD11b (M1/70), anti-I-A/I-E (M5/114.15.2), anti-CD103 (2E7), anti-CD11c (N418). The following antibodies were used to stain for IECs: anti-EpCam (G8.8) and anti-CD284 (TLR4; SA15-21). The following antibodies were utilized to stain for B10 cells: anti-CD1d (CD1.1; 1B1), anti-CD5 (53–7.3), anti-B220/CD45R (RA3-6B2), and anti-CD19 (1D3/CD19). For intracellular/intranuclear staining, the Foxp3/Transcription Factor Staining Set (eBioscience) was utilized when staining for anti-IL-17F (9D3.1C8), anti-IL-17A (TC11-18H10.1), anti-Foxp3 (150D) anti-TNFα (MP6-XT22), anti-IL-22 (Poly5164), anti-IL-10 (JES5-16E3), and anti-ZO-1 (R26.4C, eBioscience, San Diego, CA). Samples were acquired on an BD LSRII or BD FACSymphony (BD, San Jose, CA) and analyzed with FlowJo software (BD).

### 16S metagenomic sequencing library preparation

DNA was isolated from intestinal fecal content from each of the different regions using the QIAamp DNA Stool Isolation kit and Qiacube from Qiagen per manufacturer’s recommended protocol. A 16S rRNA library was prepared as follows. The PCR reaction contained microbial genomic DNA (5 ng/µl in 10 mM Tris, pH 8.5), 1 µM 16S Amplicon Forward primer (5′-TCG TCG GCA GCG TCA GAT GTG TAT AAG AGA CAG CCT ACG GGN GGC WGC AG-3′), 1 µM 16S Amplicon Reverse primer (5′-GTC TCG TGG GCT CGG AGA TGT GTA TAA GAG ACA GGA CTA CHV GGG TAT CTA ATC C-3′), 2  ×  KAPA HiFi HotStart ReadyMix (Roche Kapa Biosystems, Wilmington, MA). PCR program parameters were initial denaturation at 95 °C for 3 min, 25 cycles of 95 °C for 30 s, 55 °C for 30 s, and 72 °C for 30 s and then a final extension of 72 °C for 5 min. Next, the Index PCR was prepared using the Nextera XT Index Kit, PCR Clean-up, and Illumina MiSeq sequencing was performed following manufacturer’s protocol (Illumina, San Diego, CA). Samples were processed plug-ins provided in the qiime2 [7] pipeline. Briefly, paired end reads were joined using vsearch [39], then quality-filtered with a minimum PHRED-score of 4. The joined reads were denoised using deblur [3], producing a feature table of amplicon sequence variants (ASVs). Next, the ASVs were assigned to taxonomy using a naïve bayes classifier [6]. The classifier was trained on the sequenced region of the 16-S gene (V3-V4), using SILVA [6] v128, 99% OTU sequences. ASVs were aligned using MAFFT [21], then using only phylogenetically informative positions, an unrooted tree was constructed using FASTTree [36]. The unrooted tree and ASV feature table were imported in R and the package phyloseq [29] was used to first collapse the feature table to level of species, then calculate multiple alpha diversity metrics (Observed Richness, Simpson’s diversity index, Shannon’s diversity index) and beta diversity metrics (Jaccard distance, Bray–curtis distance, unweighted Unifrac distance and weighted Unifrac distance). PCoA plots were then generated from the beta diversity metrics. Differential abundance of taxa was assessed using R package DESeq2 [26]. Taxa were considered to be differentially abundant at an FDR of 0.05. The metagenomics for the microbiome sequencing was uploaded to Bioproject with accession number PRJNA700677.

### Statistics

Flow cytometry analyses and microbiome indexes were assessed by 2-way ANOVA with Uncorrected Fisher’s LSD. The correlation graphs of immune cell parameters to microbiome phyla abundances were performed and Pearson r values (Additional files 9, 10: Tables S1, S2) were calculated in Graph Pad Prism version 9.1.0.

## Results

### IEC TLR4 expression decreases after anastomosis

**Fig. 2:**
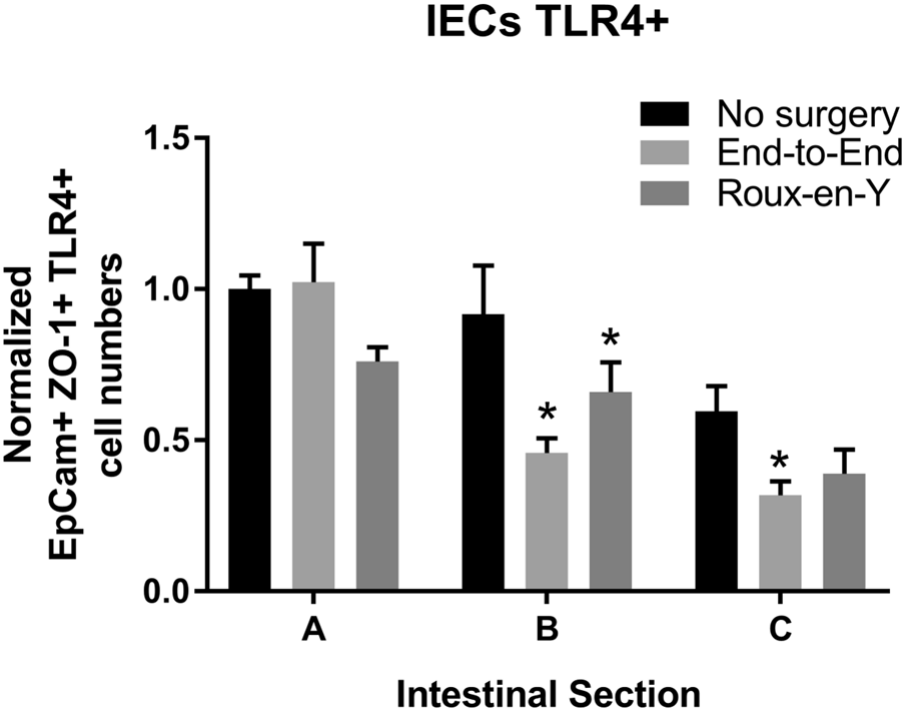
Decrease in intestinal epithelial cells (IECs) expressing TLR4^+^  cells in distal duodenum after Roux-en-Y anastomosis. Intestinal mucosal cells were harvested 3 days post-operatively and stained extracellularly with EpCam and TLR4 and intracellularly with ZO-1. Samples were then run on either a BD LSRII or Symphony. Normalized TLR4^+^  IECs (EpCam +  ZO-1^+^). *p  ≤  0.05–no surgery, n  =  7–9

From two murine surgical models, Roux-en-Y and end-to-end anastomoses (Fig. 1), we examined the TLR4 expression of EpCam^+^ ZO-1^+^ IECs by flow cytometry. We observed that the numbers of EpCam^+^ ZO-1^+^ TLR4^+^ IECs were decreased significantly after Roux-en-Y and end-to-end anastomoses within the B segment and in the C segment of end-to-end anastomosis in comparison to the respective no surgery control (Fig. 2).

### Microbial population shifts after anastomosis

**Fig. 3:**
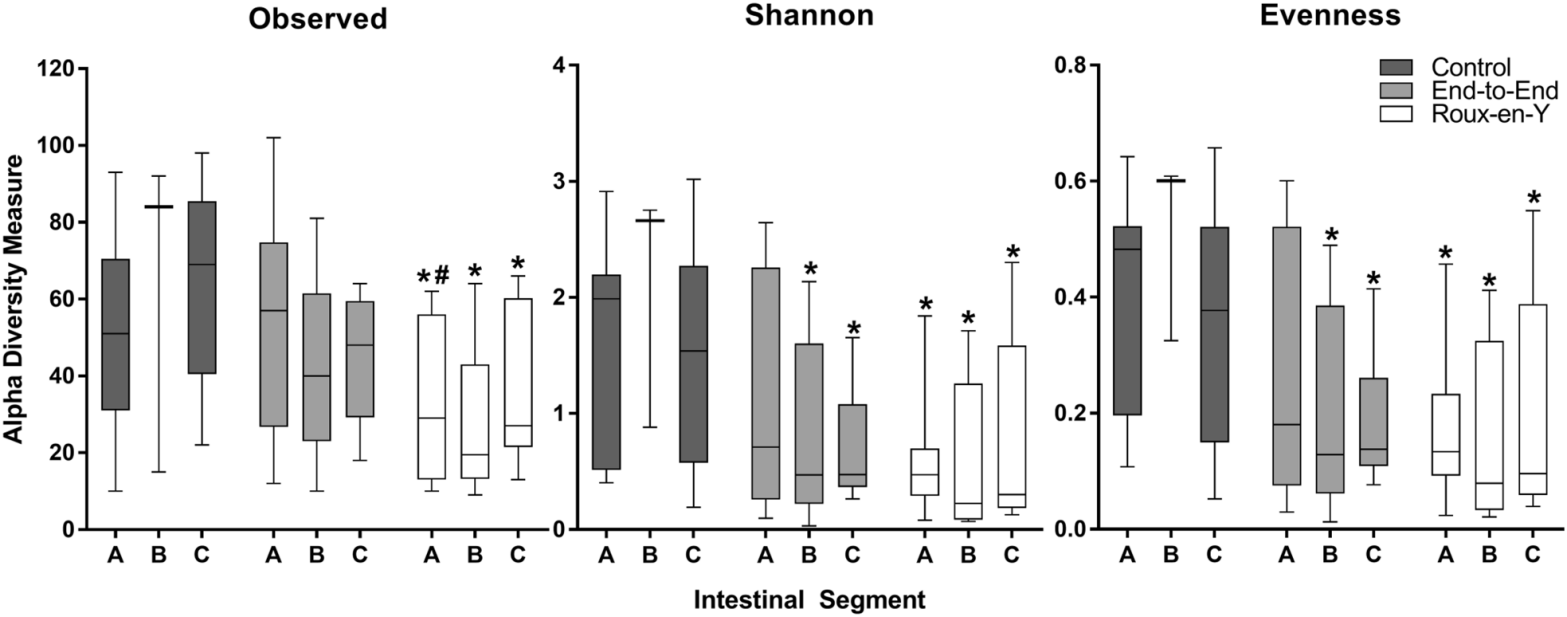
Microbiome diversity decreases after anastomosis surgery. Observed, Shannon, and Evenness diversity measures were assessed from MiSeq sequencing and qiime bioinformatics analysis

**Fig. 4:**
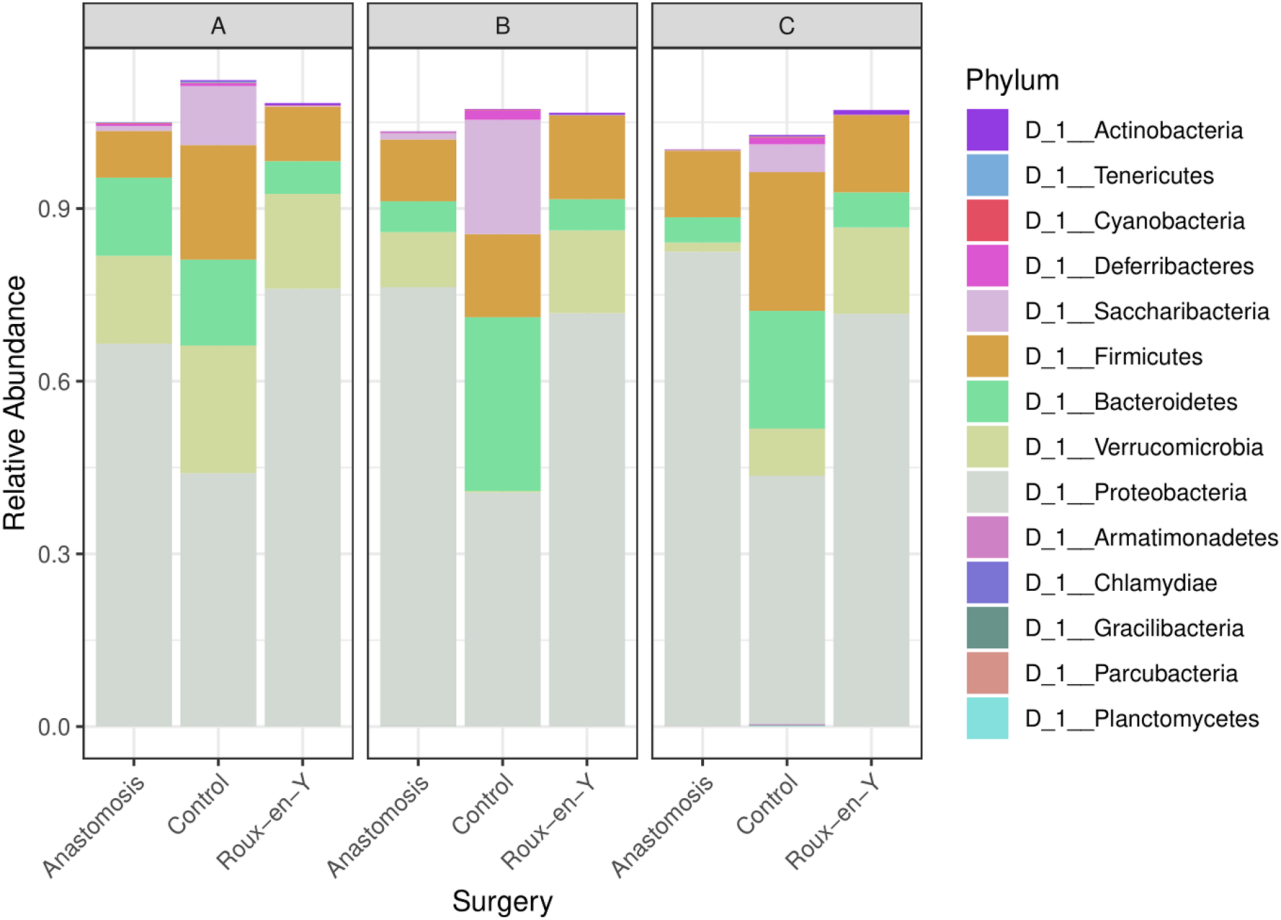
Relative abundance of Firmicutes and Bacteroidetes phylums decreased while Proteobacteria phylum increased after either anastomotic surgeries. Relative abundance composition of the intestinal microbiome phylum for each intestinal segment (top heading) within each surgery group (x-axis label)

The alpha diversity microbiome analyses showed a significant decrease in Observed, Shannon, and Evenness indices for all intestinal segments in the Roux-en-Y compared to the no surgery control (Fig. 3). Also, Roux-en-Y segment A was significantly different to the end-to-end anastomosis segment A in the Observed indices. The B and C segments after end-to-end anastomosis were only significant to no surgery control in the Shannon and Evenness indices (Fig. 3). When the relative abundances were evaluated, there was a noticeable decrease in major phyla *Firmicutes, Bacteroidetes*, and *Saccharibacteria* and an increase in *Proteobacteria* in both of the end-to-end and Roux-en-Y anastomosis surgery groups compared to no surgery control (Fig. 4). There were also significant changes seen in the bacteria order and class between the surgery groups and no surgery control (Additional file 1: Figure S1). Specifically, *Bacteroidia, Clostridia*, and *Deltaproteobacteria* class decreased, while *Gammaproteobacteria* class increased in abundance for the surgery groups (Additional file 1: Figure S1A). *Bacteroidales, Clostridiales*, and *Desulfovibrionales* order decreased, while *Enterobacteriales* order increased in abundance for the surgery groups (Additional file 1: Figure S1B).

### *Th17, iNKTh17, iNKT IL-10* + *, and Treg cells increase after anastomosis*

**Fig. 5:**
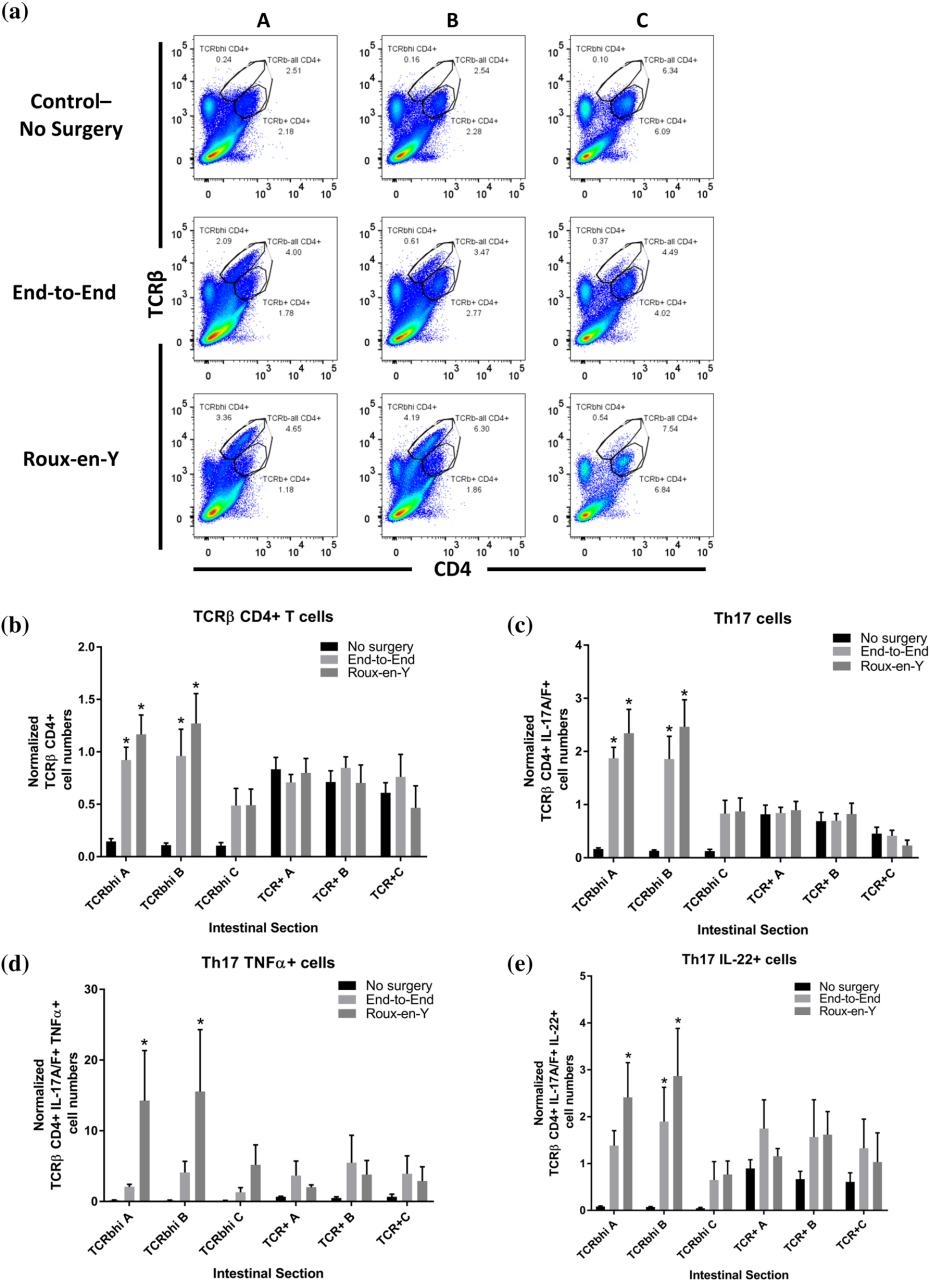
Increase in TCRβ^hi^ Th17 cells after end-to-end and Roux-en-Y anastomosis surgery. Intestinal mucosal cells were harvested 3 days post-operatively and stained extracellularly with TCRβ and CD4 and intracellularly with IL-17A, IL-17F, IL-22, and TNFα. Samples were then run on either a BD LSRII or Symphony. **A** Dot plots showing increased TCRβ expression after both anastomotic procedures from surgical segments A, B, and C. **B** Normalized TCRβ CD4^+^ T cell numbers. **C** Normalized Th17 cell numbers (TCRβ^+^ CD4^+^ IL-17A^+^ IL17F^+^). **D** Normalized Th17 cells that are TNFα^+^ or **E** IL-22^+^. *p  ≤  0.05–no surgery, n  =  5–7

**Fig. 6:**
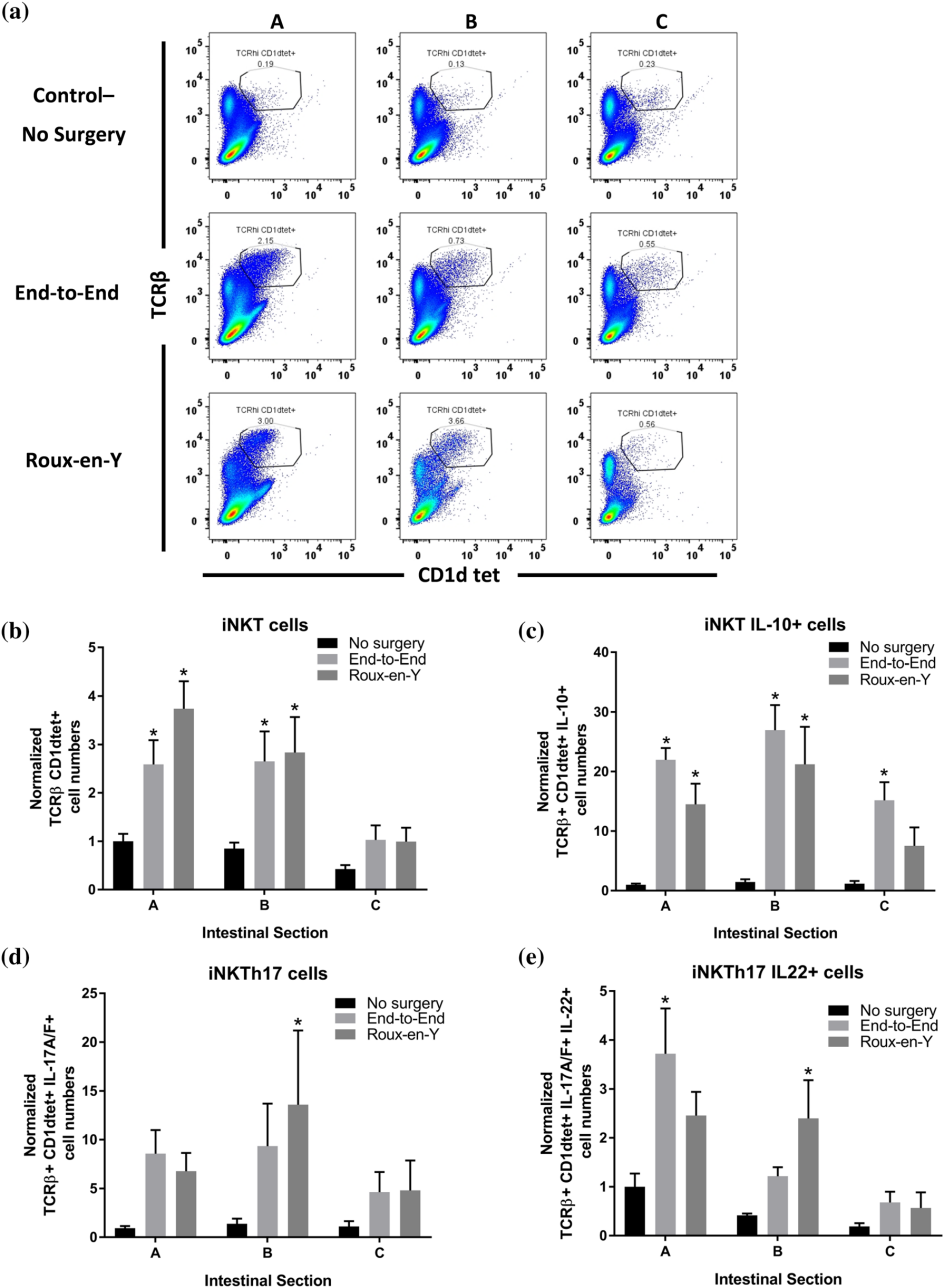
Increase in iNKT cells expressing IL-10 and IL-22 after end-to-end and Roux-en-Y anastomoses. Intestinal mucosal cells were harvested 3 days post-operatively and stained extracellularly with TCRβ and CD1dtet and intracellularly with IL-10, IL-22, IL-17A, and IL-17F. Samples were then run on either a BD LSRII or Symphony. **A**, **B** Significant increase in normalized iNKT (TCRβ^+^ CD1dtet^+^) cell numbers in distal duodenum. **C** Normalized cell numbers showing increased IL-10  +  expression in iNKT cells after both anastomotic surgeries. **D** Normalized iNKT cells that are IL-17A^+^ and IL-17F^+^ and then **E** iNKTh17 cells were gated for IL-22^+^ cells. *p  ≤  0.05–no surgery, n  =  5–8, except IL-10 and IL17A/F, n  =  8–10

**Fig. 7:**
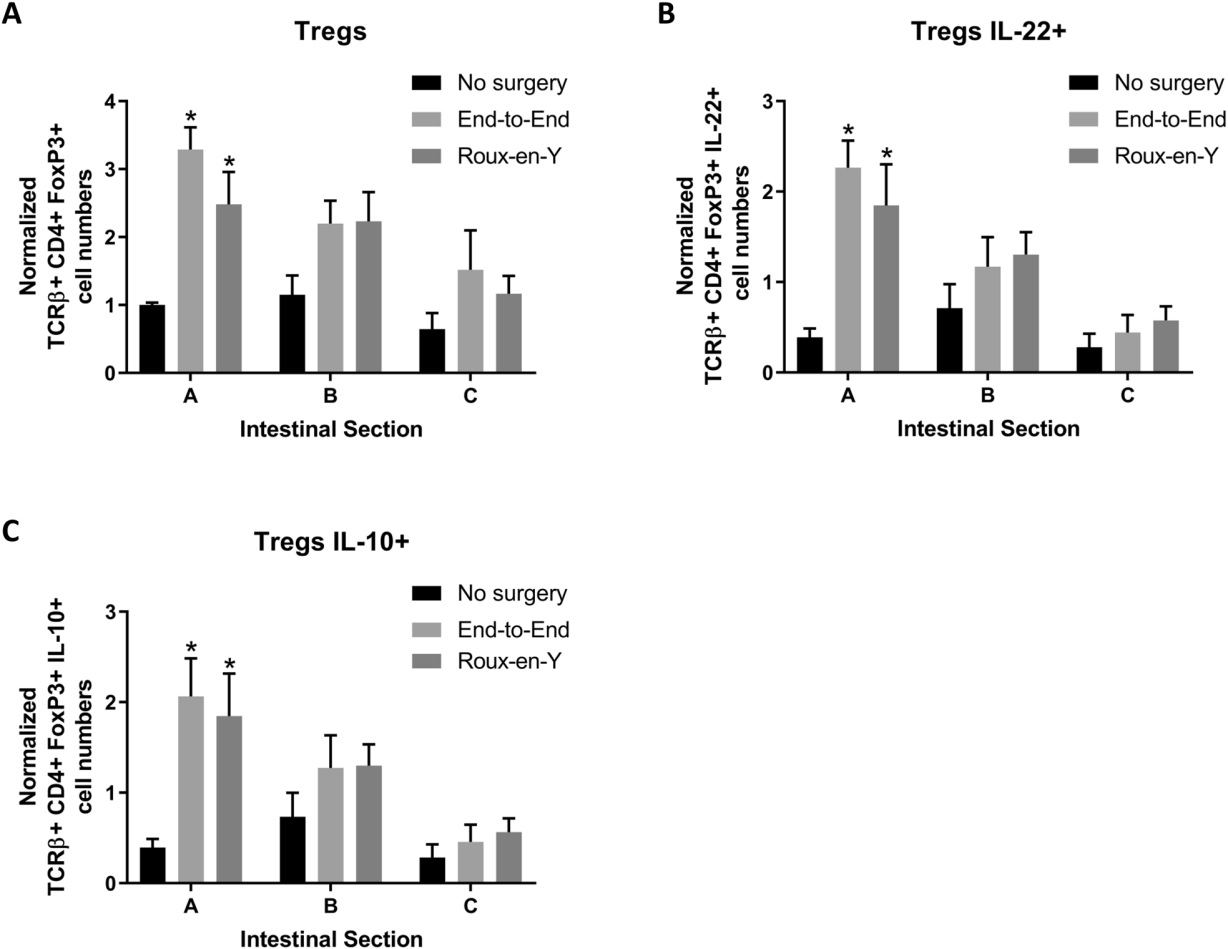
Increase in Treg cells expressing IL-10 and IL-22 after end-to-end and Roux-en-Y anastomoses. Intestinal mucosal cells were harvested 3 days post-operatively and stained extracellularly with TCRβ and CD4 and intracellularly with FoxP3, IL-10, and IL-22. Samples were then run on either a BD LSRII or Symphony. **A** Significant increase in normalized Treg (TCRβ^+^ CD4^+^) cell numbers and Treg cells that are **B** IL-22^+^ or **C** IL-10^+^. *p  ≤  0.05–no surgery, n  =  6, 7

After both Roux-en-Y and end-to-end anastomoses, we observed that the expression of TCRβ increased for both T cells and iNKT cells (Figs. 5A, 6A) in segments A and B. This increased TCRβ^hi^ CD4^+^ T cell population (Fig. 5B) were IL-17A^+^ IL-17F^+^ indicating an increase in Th17 cells (Fig. 5C). Of the TCRβ^hi^ Th17 cell population, there was a significant increase in TNFα^+^ Th17 cells after Roux-en-Y anastomosis in segments A and B (Fig. 5D). Both anastomoses had an increase in IL-22^+^ TCRβ^hi^ Th17 cells in segments A and B (Fig. 5E). iNKT cell numbers increased in anastomosis segments A and B and were TCRβ^hi^ (Fig. 6B). A significantly large percentage of iNKT cells had an increase in IL-10^+^ expression (Additional file 2: Figure S2A, Fig. 6C). Additionally, we observed a 13% increase in iNKT cells that were expressing IL-22^+^ (Additional file 2: Figure S2B), suggesting an iNKTh17 subset. Therefore, we further gated the iNKT cells with IL-17A/F and indeed we observed a trending increase in iNKTh17 and iNKTh17 IL-22^+^ cells with significance reached in the end-to-end segment A and Roux-en-Y segment B in comparison to the respective no surgery controls (Fig. 6D, E). Alternatively, the Treg population was TCRβ^+^ but was not high in TCRβ (Additional file 3: Figure S3). The Treg cells were increased after both anastomoses surgeries with significance seen most greatly in segment A (Fig. 7A). These Treg cells were also significantly expressing IL-10^+^ and IL-22^+^ in segment A (Fig. 7B, C; Additional file 4: Figure S3).

### ***A novel CD11b***^***hi***^*** CD103***^***mid***^*** DC population emerges after anastomosis***

**Fig. 8:**
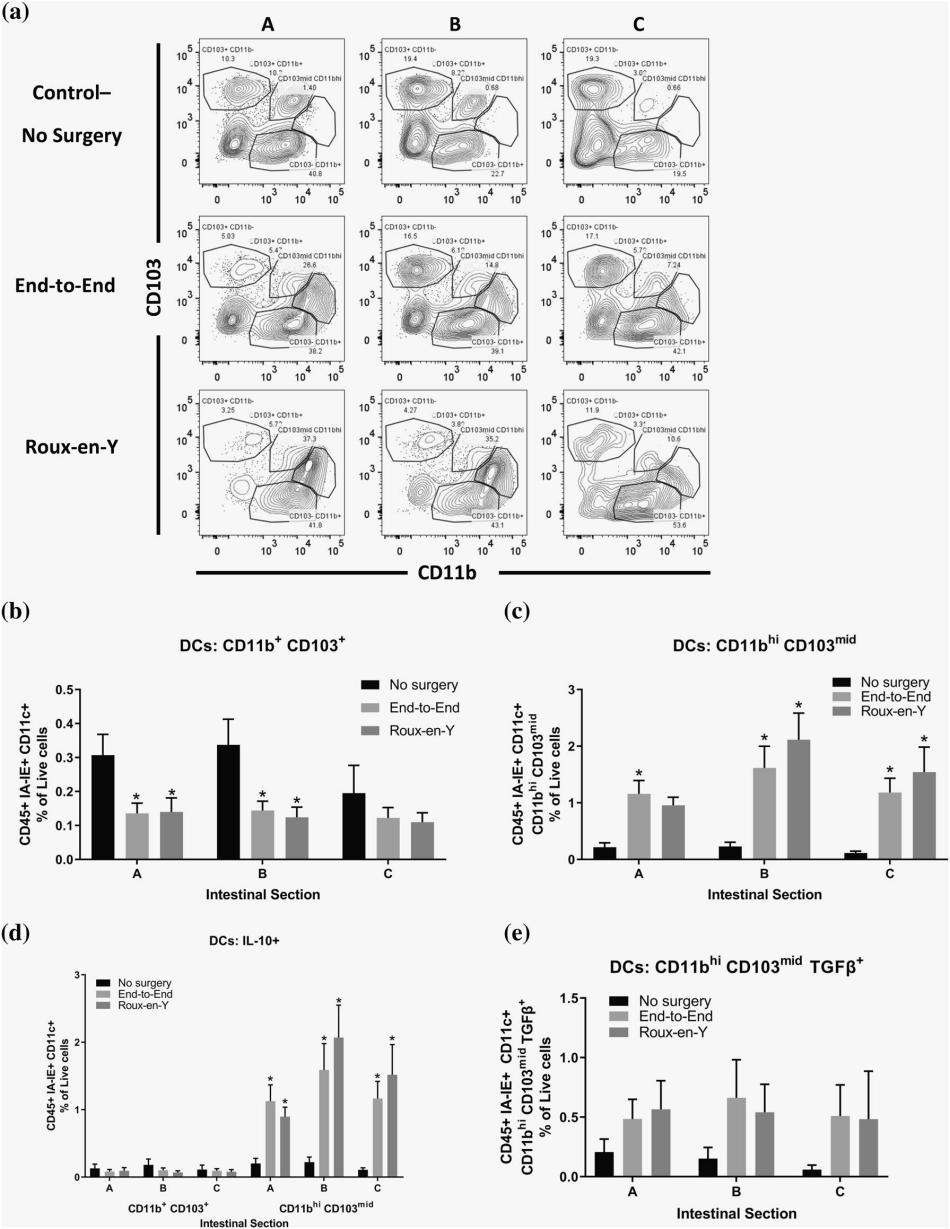
A new population of CD11b^hi^ CD103^mid^ DCs emerges after both end-to-end and Roux-en-Y anastomosis and are expressing IL-10. Intestinal mucosal cells were harvested 3 days post-operatively and stained extracellularly with CD45, IA-IE, CD110, CD11b, and CD103 and intracellularly with IL-10. Samples were then run on either a BD LSRII or Symphony. **A** Dot plot representation and **B** % of live cell numbers of CD11b^+^ CD103^+^ and **C** CD11b^hi^ CD103^mid^ DC (CD45^+^ IA-IE^+^ CD110^+^) populations. **D** Expression of IL-10 in CD11b CD103 DC populations. **E** Percent of live cell numbers of CD11b^hi^ CD103^mid^ DCs that are TGFβ^+^ *p  ≤  0.05–no surgery, n  =  6–9

We observed a decreased shift in CD11b^+^ CD103^+^ DCs to a new population subset of CD11b^hi^ CD103^mid^ DC population after end-to-end and Roux-en-Y anastomoses (Fig. 8A, B, C). This new subset of DCs had an increase in the expression of IL-10 in all three segments, while the CD11b^+^ CD103^+^ populations had a trending decrease (Fig. 8D). TGFβ expression in DC subsets were also evaluated but there were no significant differences between the intestinal segments or between the control and surgery groups, although there was a trending increase (Fig. 8E).

### Direct correlations between microbiome phlya and immune cell populations

**Fig. 9:**
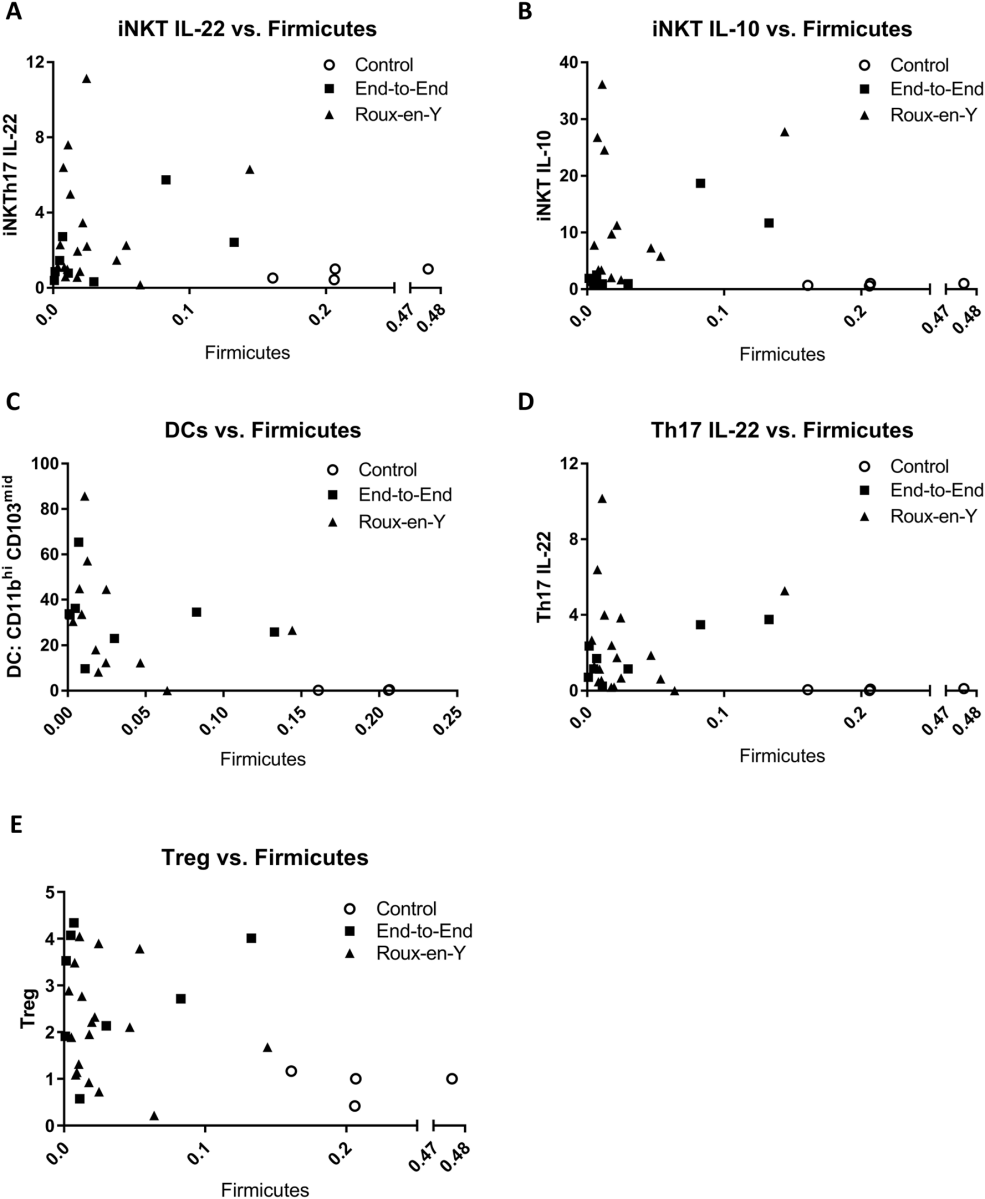
Immune cell populations correlate to Firmicutes with distinct patterns based on surgical segments. Correlation graphs of **A** iNKT (TCRβ^+^ CD1dtet^+^) IL-22^+^ cells, **B** iNKT IL-10^+^ (significant r value of 0.8186 in segment B and 0.719 for Segment C), **C** CD11b^hi^ CD103^mid^ DCs (CD45^+^ IA-IE^+^ CD110^+^), **D** Th17 (TCRβ^hi^ CD4^+^ IL-17A^+^ IL17F^+^) and **E** Treg (TCRβ^+^ CD4^+^ FoxP3^+^) (significant r value of 0.0428 for segment C) versus Firmicutes within surgical segments. Pearson correlation coefficient (r) are found in Additional file 9: Table S1

**Fig. 10:**
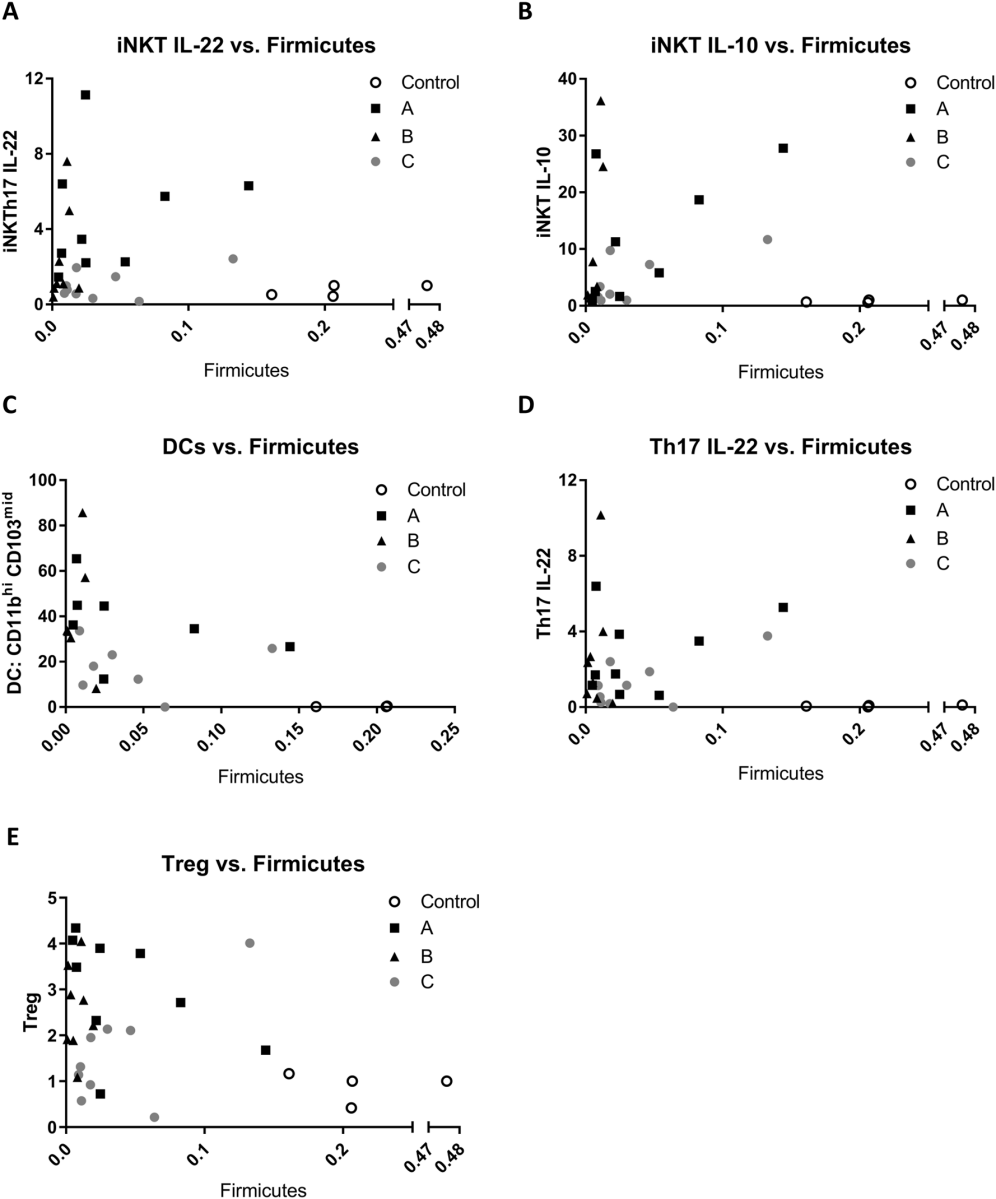
Immune cell populations correlate to Firmicutes with distinct patterns based on either anastomotic surgery. Correlation graphs of **A** iNKT (TCRβ^+^ CD1dtet^+^) IL-22^+^ cells, **B** iNKT IL-10^+^ (significant r value of 0.8138 for end-to-end anastomosis), **C** CD11b^hi^ CD103^mid^ DCs (CD45^+^ IA-IE^+^ CD110^+^), **D** Th17 (TCRβ^hi^ CD4^+^ IL-17A^+^ IL17F^+^) (significant r value of 0.8151 for end-to-end anastomosis) and **E** Treg (TCRβ^+^ CD4^+^ FoxP3^+^) versus Firmicutes within surgery type. Pearson correlation coefficient (r) are found in Additional file 10: Table S2

When the major phyla *Firmicutes, Bacteroidetes*, and *Proteobacteria* were correlated to immune cell populations iNKT IL-22^+^, iNKT IL-10^+^, DC CD11b^hi^ CD103^mid^, Th17 IL-22^+^, and Tregs. There were consistent, correlative shifts that were observed in comparison to the control. The correlation graphs reveal the direct association of increases in iNKTs, DCs, Th17, and Treg cells to decreases in *Firmicutes* and *Bacteroidetes* and an increase in *Proteobacteria* within the end-to-end and Roux-en-Y surgery groups (Fig. 9, Additional files 5, 6: Figures S4, S5). Additionally, when only the surgical segments were assessed, we observed that the change in bacteria phyla were consistent across all surgery segments with the highest numbers of iNKTs, DCs, Th17 and Treg cells being in segment A and decreased as we moved distally to the segment C (Fig. 10, Additional files 7, 8: Figures S6, S7). Overall, there was a correlative significance observed in iNKT IL-10 vs. *Firmicutes* in segments B and C (Fig. 10, Additional file 9: Table S1) and the end-to-end anastomosis had correlative significance in the iNKT IL-10 vs. *Firmicutes* and the Th17 vs. *Firmicutes* correlations (Fig. 9, Additional file 10: Table S2). The Treg cells had a correlative significance to *Bacteroidetes* and *Proteobacteria* in segment A and to *Firmicutes* in segment C (Fig. 10, Additional file 9: Table S1).

## Discussion

This current study further identifies the immune cell and microbiome shifts that occur after small intestinal anastomosis, in comparison to the homeostatic non-surgical control. We did not perform a sham-operated control as we felt the non-surgical control offered a better perspective on the presurgical normal microbiome and immune cell population environment to make comparisons to, however, we do recognize that a sham-operated control could have added more rigor to these studies, and we will consider doing them in our future work. Overall, we observed a decrease in microbiome diversity which included decreases in the major phyla *Firmicutes, Bacteroidetes*, and *Saccharibacteria* but then an increase in *Proteobacteria* after anastomosis. We found that there were increases in Th17 and iNKTh17 cell populations and that these Th17 and iNKTh17 cells had a higher expression of TCRβ, while the increased Treg populations had homeostatic expression levels of TCRβ. Additionally, these Treg populations also had an increase in IL-10 and IL-22, which are necessary for mucosal wounding healing [25]. Evaluating the DC populations after anastomosis revealed an increase in a subpopulation that was CD11b^hi^ CD103^mid^, while the conventional CD11b^+^ CD103^+^ DCs decreased. The CD11b^hi^ CD103^mid^ DCs were expressing IL-10. The increases in iNKTs, DCs, Th17, and Treg cells correlated to decreases in *Firmicutes* and *Bacteroidetes* phyla and an increase in the *Proteobacteria* phylum within the end-to-end and Roux-en-Y anastomoses surgery groups.

Studies involving germ-free mice have demonstrated that there is an impaired rate of intestinal epithelial cell migration, indicating the necessity of commensal bacteria for effective migration during wound healing [2]. The pattern-recognition receptor, TLR4 specifically detects the conserved molecular product lipopolysaccharide (LPS) on microorganisms [37]. The key functions of the TLR family are to act as sensors of microbial infection, initiate inflammatory and immune responses, and to aid in mucosal epithelial healing and homeostasis [37]. We specifically observed that the expression of TLR4 decreased after anastomotic surgery (Fig. 2). Interestingly, if there is not proper activation of TLRs on epithelial cells, there will be abnormalities in the intestinal epithelial homeostasis and production of cytokines and heat-shock proteins. If there is a defect in the assembly and release of these factors after intestinal injury, the detection and protection of commensals is impeded [37]. Therefore, this leads to a dysregulated interaction between commensals and TLRs resulting in chronic inflammation and tissue damage. So, a critical balance between the activation of TLRs by commensals and the protective action induced by TLRs will impact whether proper homeostasis can be maintained, or mucosal injury (including by surgery) can be properly healed. How TLR involvement and inflammation affect the microbiome environment could be further explored by comparing the effects of intraperitoneal administration of TLR4 agonist LPS from *Escherichia coli* versus administration of TLR4 antagonist LPS from cyanobacteria *Rhodobacter sphaeroides* after anastomotic surgery [51]. However, this exceeds the scope of the current study.

IBD patients are associated with a lower abundance of *Firmicutes* and higher abundance of *Proteobacteria* and *Bacteroidetes* [2, 28]. Mice that have undergone ileocecal resection (ICR) plus a single antibiotic injection at surgery, have decreased phylogenetic diversity by 7 days post-operative. Prior to ICR, *Firmicutes* and *Bacteroidetes* were the predominant strains in the jejunum, but then only *Firmicutes* remained the dominant population 28 days after ICR, whereas *Bacteroidetes* was 0.01% of abundance and there was an increase in *Proteobacteria* [12]. Another study conducted 75% small bowel resection (SBR) in piglets and showed an increase in *Firmicutes* and a decrease in *Bacteroidetes* and *Proteobacteria* 2 and 6 weeks post-operative [23]. Another study performed 50% proximal SBR in mice and found that there was little to no difference in microbiota diversity between the SBR mice and sham mice that had a transection and anastomosis 7 days post-operative [48]. They did not however compare the sham mice to non-operated mice. In our anastomotic study, we observed significant decrease in microbiome diversity and in major phyla *Firmicutes, Bacteroidetes*, and *Saccharibacteria* but an increase in *Proteobacteria* in both of the end-to-end and Roux-en-Y anastomosis surgery groups compared to non-operative control. The differences we observed in the microbiome diversity in comparison to the forementioned studies could be due to timing of post-operative fecal collection (our study collected at day 3 vs. others at days 7, 14, and 42), type of surgery performed, and the species. Therefore, our study evaluates an acute time period that is necessary for the establishment of proper healing or by which the timing when most anastomoses start to fail [43]. Moreover, we correlated these diversity changes to the response of the immune cells at day 3. We correlated the decrease in *Firmicutes* after anastomosis surgery to an increase in Th17 IL-22^+^ cells, with the greatest increases seen in the proximal segments A and B and a less of an increase in the distal segment C ileal region (Fig. 10D). This contrasts to how segmented filamentous bacteria from the *Firmicutes* phylum, are able to bind to the epithelial surface of the ileum and penetrate the mucus layer [25]. They then can induce the differentiation of CD4^+^ T cells to Th17 cells [19, 25]. Previous studies have also shown that 17 strains of *Clostridia* and *B. fragilis* support the induction and proliferation of Tregs that produce IL-10 [9, 41]. Interestingly, we observed a contrasting correlation between the *Clostridia* and *Bacteroidia* classes, in that after anastomotic surgery they decreased while IL-10^+^ Treg numbers increased. These differences again could be due to the timing of post-operative collection and what type of surgery was performed.

Post-operatively, we observed that the Th17, and iNKTh17 populations had a higher expression of TCRβ than control non-surgical mice. TCR upregulation can occur when the cells are preparing for mitosis or even independent of cell division [27, 46]. It has been proposed that enhancement of TCR expression level overall is directly linked to T cell activation, this is so that the T cell sensitivity is elevated to peptide antigen [45]. Lastly, ceramide is known to induce TCR upregulation [45]. After mesenteric ischemic/reperfusion injury, ceramide concentration levels were increased in intestinal vasculature and coating the membranes of bacteria [17]. Ceramide is known to be involved in the host response to bacterial infections [17, 54, 55]. Therefore, the increased TCR expression that we observed after anastomotic surgery on Th17 and iNKTh17 cell populations could be due to long stimulation and a change in bacterial populations and ceramide levels, but this awaits further exploration.

Characterizing conventional DCs (cDCs) has led to intense debates and broad investigations. The issue is that several of the markers used to define DCs are also expressed by macrophages, including CD45, CD11c, CD11b, and MHC class II (IA–E). However, macrophages do not express the integrin α_E_ chain CD103 [5, 35, 38, 50]. CD103^+^ DCs are typical migratory cells in the lamina propria that have mainly been divided into two populations, CD11b^+^ CD103^+^ and CD11b^−^ CD103^+^ cDCs [35]. For this reason, it was unexpected to us to find an increase in a new cDC population, CD11b^hi^ CD103^mid^, within all of the intestinal segments after anastomosis surgery. This novel population appeared to be a shift away from the CD11b^+^ CD103^+^ cDC population as these cells decreased significantly after surgery. Now it is unknown whether there are distinct precursors for the CD11b^+^ CD103^+^ and CD11b^−^ CD103^+^ cDCs or even what signals they obtain from the intestinal environment that leads to their final differentiation state [35]. Therefore, our data indicates that a post-operative environment leads to a new CD11b^hi^ CD103^mid^ DC differentiated population. Additionally, of the cDC populations, the CD11b^hi^ CD103^mid^ DCs were expressing IL-10, an anti-inflammatory cytokine. It is known that activated CD11b^+^ DCs in the Peyer’s patches produce higher levels of IL-10 than splenic DCs and are better able to activate CD4^+^ T cells to produce higher levels of IL-4 and IL-10 [10, 11]. CD103^+^ DCs, but not CD103^−^ DCs, from the lamina propria are also known for being able to induce FoxP3 in naïve T cells better than splenic DCs in the presence of exogenous TGFβ [10, 11]. Our post-operative results showed an increase in IL-10^+^ CD11b^hi^ CD103^mid^ DCs, which then correlated with an increase in TCRβ^hi^ CD4^+^ T cells and Treg cells. These Treg cells had increased expression of IL-10 and IL-22, which are important for healing pathways [25]. Along with the IL-10^+^ Treg population increase, there was an increase in iNKT IL-10^+^ cells post-operatively. CD11c^+^ DCs can regulate iNKT cell homeostasis and activation via CD1d-dependent presentation of intestinal lipids [42]. This DC-iNKT cell crosstalk is important for controlling the bacteria and immune cell populations, such as Tregs within the intestinal compartment as mice with a CD1d conditional deletion have dysbiosis and altered immune homeostasis [42]. Importantly, Cre^+^ CD1d^fl/fl^ CD11c^Cre^ mice orally administered the lipid iNKT activator αGalCer had failed to increase Treg populations in the intestinal mesenteric lymph node whereas WT mice had increased Treg populations [42].

## Conclusions

This study defines the microbiota and immune processes that take place to during anastomotic healing. By understanding which microbiome populations that are lost and how this may affect the induction and differentiation of immune populations, we can then target post-operative therapeutic treatments that allow for suitable and accelerated healing.

## Supplementary Information

**Additional file 1: Figure S1.** Relative abundance of class and order bacterial groups change due to either anastomotic surgeries. (A) Relative abundance composition of the intestinal microbiome class or (B) order for each intestinal segment (top heading) within each surgery group (x-axis label).

**Additional file 2: Figure S2.** Expression of IL-10 and IL-22 in iNKT cells after end-to-end and Roux-en-Y anastomoses. (A) Dot plots showing increased IL-10+ expression in iNKT cells after both anastomotic surgeries. (B) Normalized iNKT cells that are IL-22^+^. * = p ≤ 0.05, n=5-8.

**Additional file 3: Figure S3.** Treg gating and expression of IL-10 and IL-22. (A) Dot plots showing Treg cells after (A) no surgery, (B) end-to-end anastomosis, and (C) Roux-en-Y anastomosis from segment A. FoxP3 vs. TCRβ dot plots (2^nd^ column from top to bottom) back gate to either TCRβ^hi^ CD4^+^, TCRβ^hi/+^ CD4^+^, or TCRβ^+^ CD4^+^ gates in the first dot plot. IL-22 and IL-10 plots are back gated to the TCRβ^hi/+^ CD4^+^ FoxP3^+^ parent plot. Representative of one experiment, n=6-7.

**Additional file 4: Figure S3.** Treg gating and expression of IL-10 and IL-22. (A) Dot plots showing Treg cells after (A) no surgery, (B) end-to-end anastomosis, and (C) Roux-en-Y anastomosis from segment A. FoxP3 vs. TCRβ dot plots (2nd column from top to bottom) back gate to either TCRβhi CD4+, TCRβhi/+ CD4+, or TCRβ+ CD4+ gates in the first dot plot. IL-22 and IL-10 plots are back gated to the TCRβhi/+ CD4+ FoxP3+ parent plot. Representative of one experiment, n=6-7.

**Additional file 5: Figure S4.** Immune cell populations correlate to Bacteroidetes with distinct patterns based on either anastomotic surgery. Correlation graphs of (A) iNKT (TCRβ+ CD1dtet+) IL-22+ cells, (B) iNKT IL-10+, (C) CD11bhi CD103mid DCs (CD45+ IA-IE+ CD110+), (D) Th17 (TCRβhi CD4+ IL-17A+ IL17F+) and (E) Treg (TCRβ+ CD4+ FoxP3+) versus Bacteroidetes within surgery type.

**Additional file 6: Figure S5.** Immune cell populations correlate to Proteobacteria with distinct patterns based on either anastomotic surgery. Correlation graphs of (A) iNKT (TCRβ+ CD1dtet+) IL-22+ cells, (B) iNKT IL-10+, (C) CD11bhi CD103mid DCs (CD45+ IA-IE+ CD110+), (D) Th17 (TCRβhi CD4+ IL-17A+ IL17F+) and (E) Treg (TCRβ+ CD4+ FoxP3+) versus Proteobacteria within surgery type.

**Additional file 7: Figure S6.** Immune cell populations correlate to Bacteroidetes with distinct patterns based on surgical segments. Correlation graphs of (A) iNKT (TCRβ+ CD1dtet+) IL-22+ cells, (B) iNKT IL-10+, (C) CD11bhi CD103mid DCs (CD45+ IA-IE+ CD110+), (D) Th17 (TCRβhi CD4+ IL-17A+ IL17F+) and (E) Treg (TCRβ+ CD4+ FoxP3+) versus Bacteroidetes within surgical segments.

**Additional file 8: Figure S7.** Immune cell populations correlate to Proteobacteria with distinct patterns based on surgical segments. Correlation graphs of (A) iNKT (TCRβ^+^ CD1dtet^+^) IL-22^+^ cells, (B) iNKT IL-10^+^, (C) CD11b^hi^ CD103^mid^ DCs (CD45^+^ IA-IE^+^ CD110^+^), (D) Th17 (TCRβ^hi^ CD4^+^ IL-17A^+^ IL17F^+^) and (E) Treg (TCRβ^+^ CD4^+^ FoxP3^+^) versus Proteobacteria within surgical segments.

**Additional file 9: Table S1.** Pearson correlation coefficients (r) for immune cell population versus phylums within anastomoses segments. Bolded r value with * have a p ≤ 0.05 and indicates a significant correlation between the immune cell population and phylum within that surgical segment.

**Additional file 10: Table 2.** Pearson correlation coefficients (r) for immune cell population versus phylums within surgery types. Bolded r value with * have a p ≤ 0.05 and indicates a significant correlation between the immune cell population and phylum within that type of surgery.

## Data Availability

Supplemental figures and tables are uploaded at https://doi.org/10.6084/m9.figshare.13645880 and link https://figshare.com/s/c485e4b8ea555cb4cd02. The metagenomics for the microbiome sequencing was uploaded to Bioproject with accession number PRJNA700677.
