# Supplementary figures and images for: Microbiome diversity declines while distinct expansions of Th17, iNKT, and dendritic cell subpopulations emerge after anastomosis surgery
Source: Gut Pathog. 2021 Aug 10;13:51. doi: 10.1186/s13099-021-00447-z (PMC8353768; doi:10.1186/s13099-021-00447-z)

Supp. Figure 1.

A.

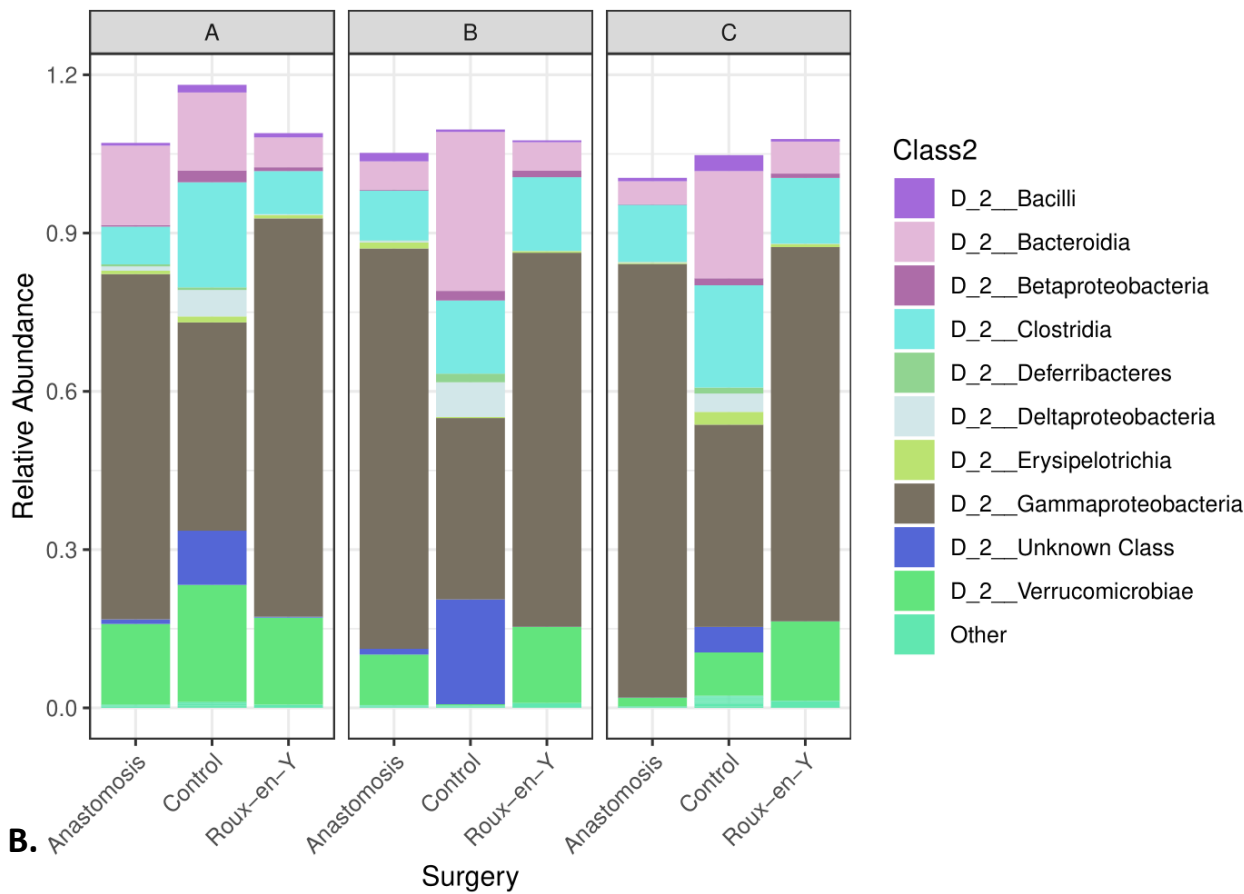

B.

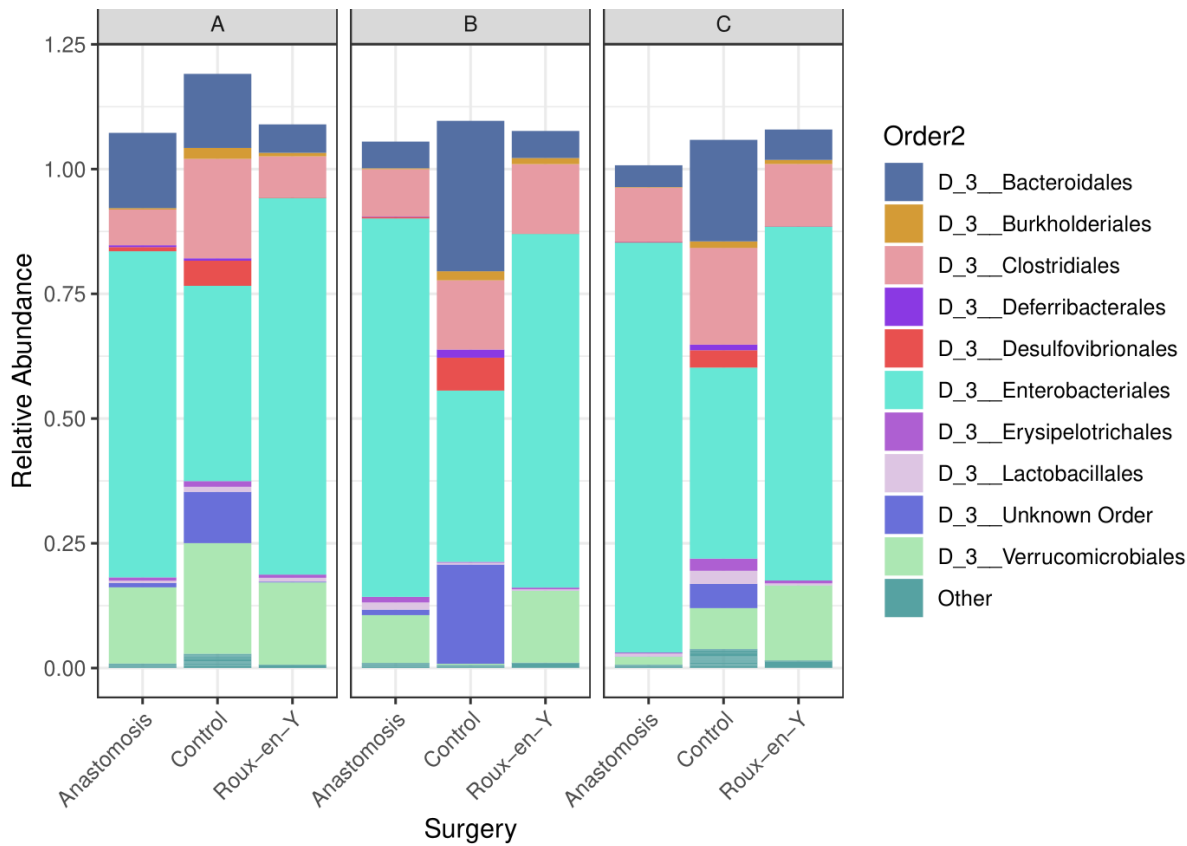

Supplement: Supplementary file 1 — Additional file 1: Figure S1. Relative abundance of class and order bacterial groups change due to either anastomotic surgeries. (A) Relative abundance composition of the intestinal microbiome class or (B) order for each intestinal segment (top heading) within each surgery group (x-axis label). [file 13099_2021_447_MOESM1_ESM.pdf]

Supp. Figure 2.

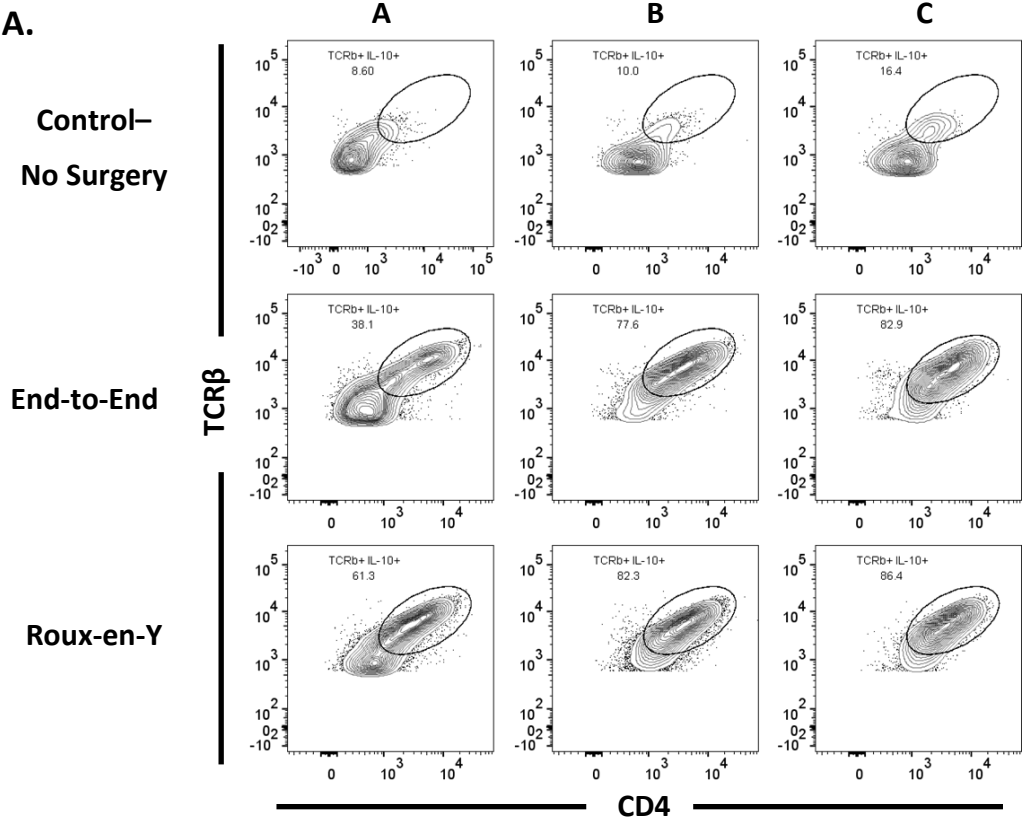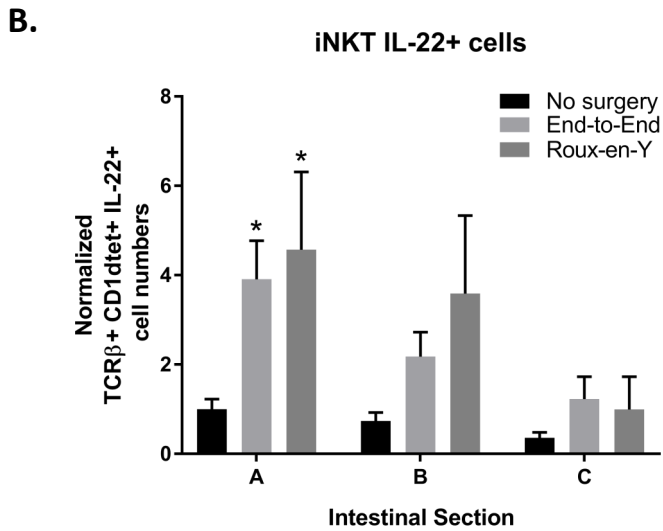

Supplement: Supplementary file 2 — Additional file 2: Figure S2. Expression of IL-10 and IL-22 in iNKT cells after end-to-end and Roux-en-Y anastomoses. (A) Dot plots showing increased IL-10+ expression in iNKT cells after both anastomotic surgeries. (B) Normalized iNKT cells that are IL-22+. * = p ≤ 0.05, n=5-8. [file 13099_2021_447_MOESM2_ESM.pdf]

Supp. Figure 3.

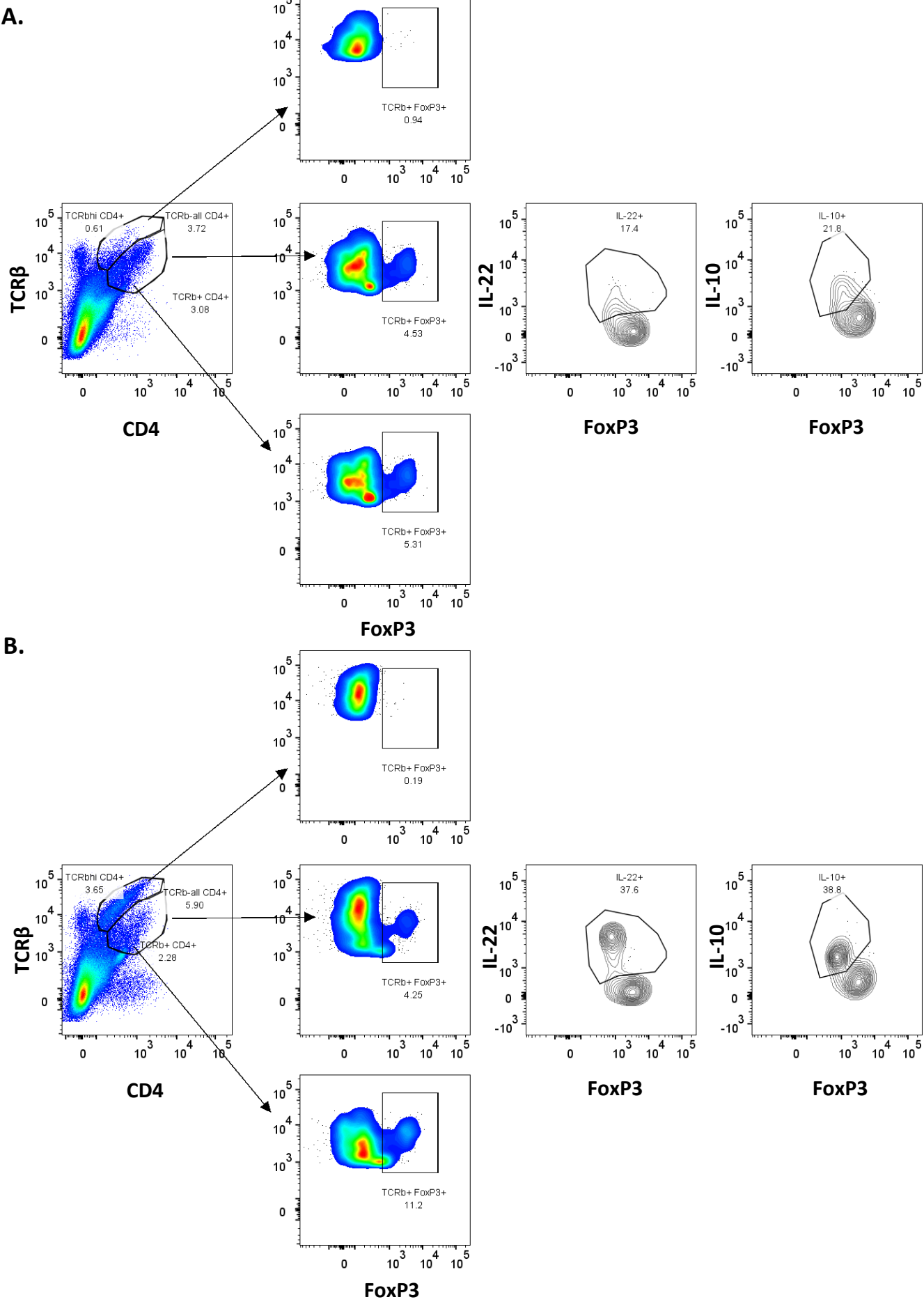

Supplement: Supplementary file 3 — Additional file 3: Figure S3. Treg gating and expression of IL-10 and IL-22. (A) Dot plots showing Treg cells after (A) no surgery, (B) end-to-end anastomosis, and (C) Roux-en-Y anastomosis from segment A. FoxP3 vs. TCRβ dot plots (2nd column from top to bottom) back gate to either TCRβhi CD4+, TCRβhi/+ CD4+, or TCRβ+ CD4+ gates in the first dot plot. IL-22 and IL-10 plots are back gated to the TCRβhi/+ CD4+ FoxP3+ parent plot. Representative of one experiment, n=6-7. [file 13099_2021_447_MOESM3_ESM.pdf]

Supp. Figure 3.

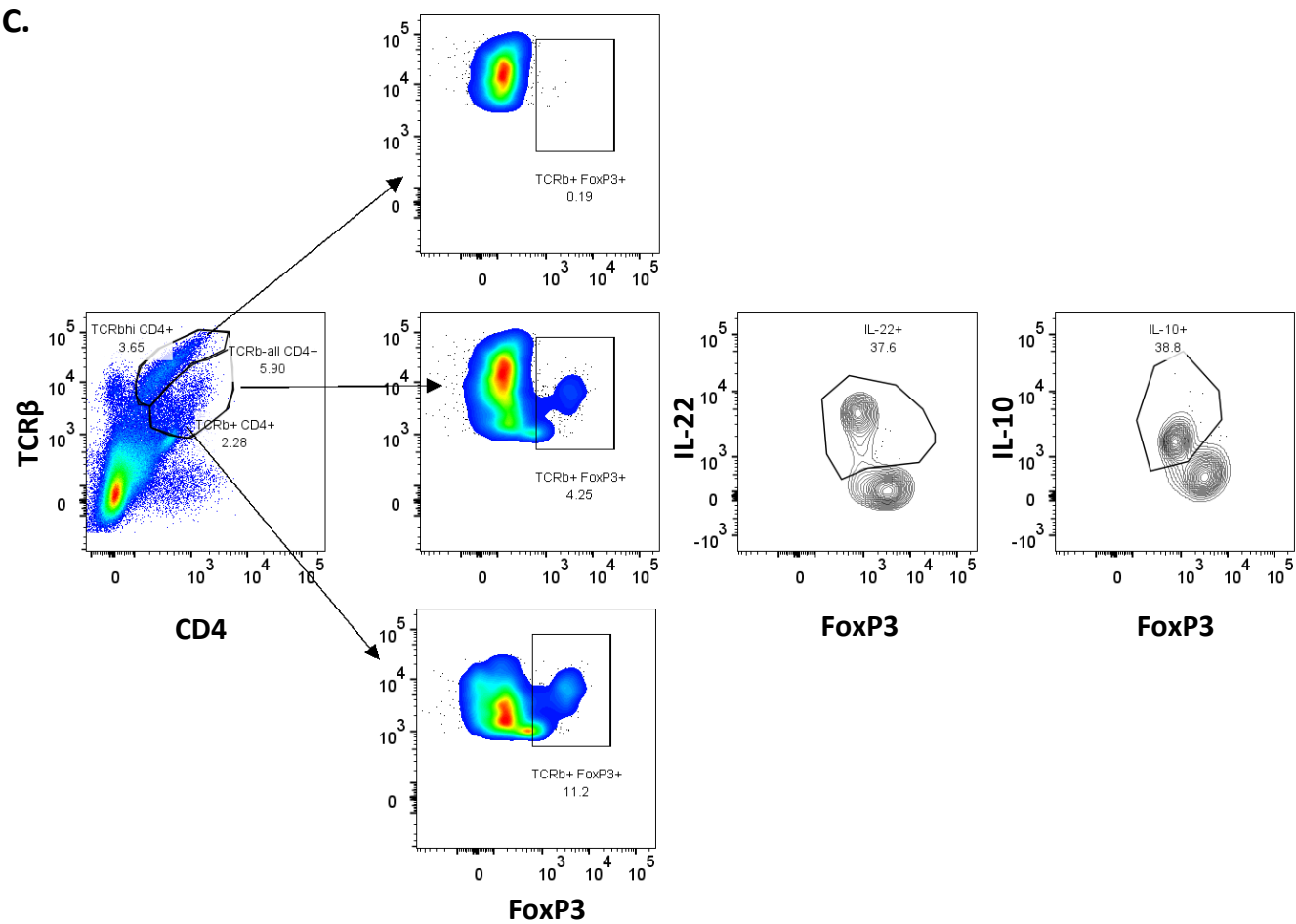

Supplement: Supplementary file 4 — Additional file 4: Figure S3. Treg gating and expression of IL-10 and IL-22. (A) Dot plots showing Treg cells after (A) no surgery, (B) end-to-end anastomosis, and (C) Roux-en-Y anastomosis from segment A. FoxP3 vs. TCRβ dot plots (2nd column from top to bottom) back gate to either TCRβhi CD4+, TCRβhi/+ CD4+, or TCRβ+ CD4+ gates in the first dot plot. IL-22 and IL-10 plots are back gated to the TCRβhi/+ CD4+ FoxP3+ parent plot. Representative of one experiment, n=6-7. [file 13099_2021_447_MOESM4_ESM.pdf]

Supp. Figure 4.

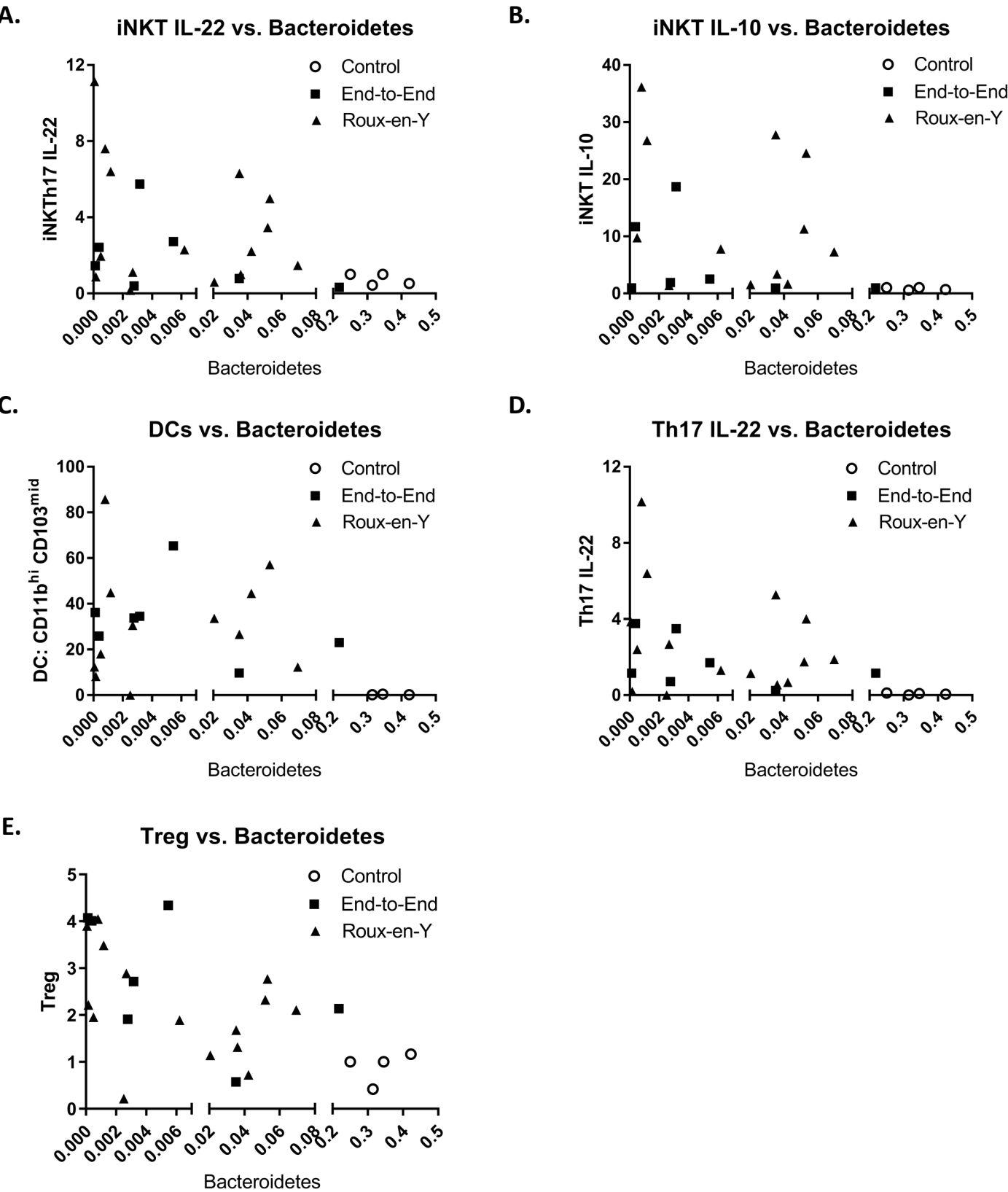

Supplement: Supplementary file 5 — Additional file 5: Figure S4. Immune cell populations correlate to Bacteroidetes with distinct patterns based on either anastomotic surgery. Correlation graphs of (A) iNKT (TCRβ+ CD1dtet+) IL-22+ cells, (B) iNKT IL-10+, (C) CD11bhi CD103mid DCs (CD45+ IA-IE+ CD110+), (D) Th17 (TCRβhi CD4+ IL-17A+ IL17F+) and (E) Treg (TCRβ+ CD4+ FoxP3+) versus Bacteroidetes within surgery type. [file 13099_2021_447_MOESM5_ESM.pdf]

Supp. Figure 5

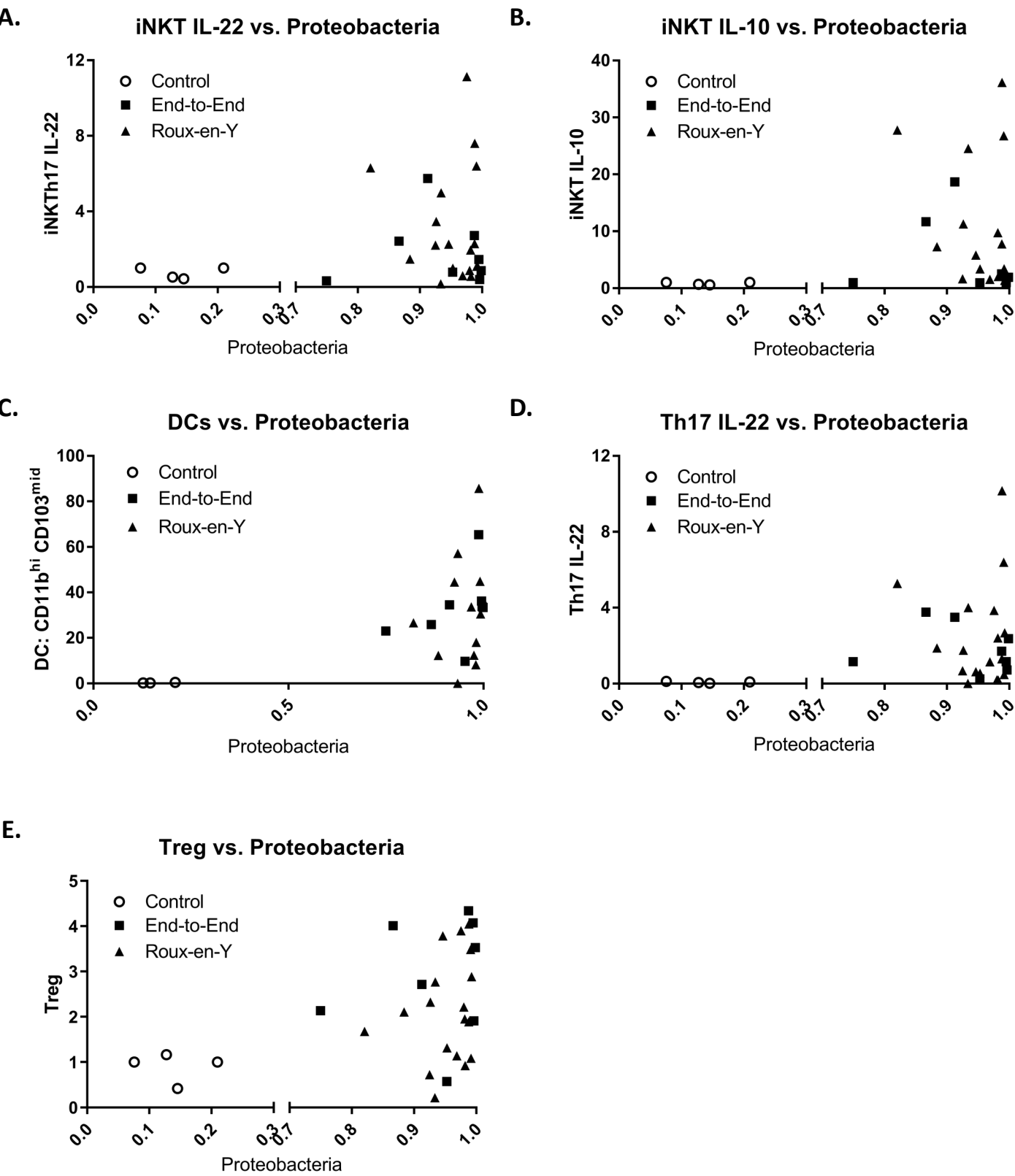

Supplement: Supplementary file 6 — Additional file 6: Figure S5. Immune cell populations correlate to Proteobacteria with distinct patterns based on either anastomotic surgery. Correlation graphs of (A) iNKT (TCRβ+ CD1dtet+) IL-22+ cells, (B) iNKT IL-10+, (C) CD11bhi CD103mid DCs (CD45+ IA-IE+ CD110+), (D) Th17 (TCRβhi CD4+ IL-17A+ IL17F+) and (E) Treg (TCRβ+ CD4+ FoxP3+) versus Proteobacteria within surgery type. [file 13099_2021_447_MOESM6_ESM.pdf]

Supp. Figure 6.

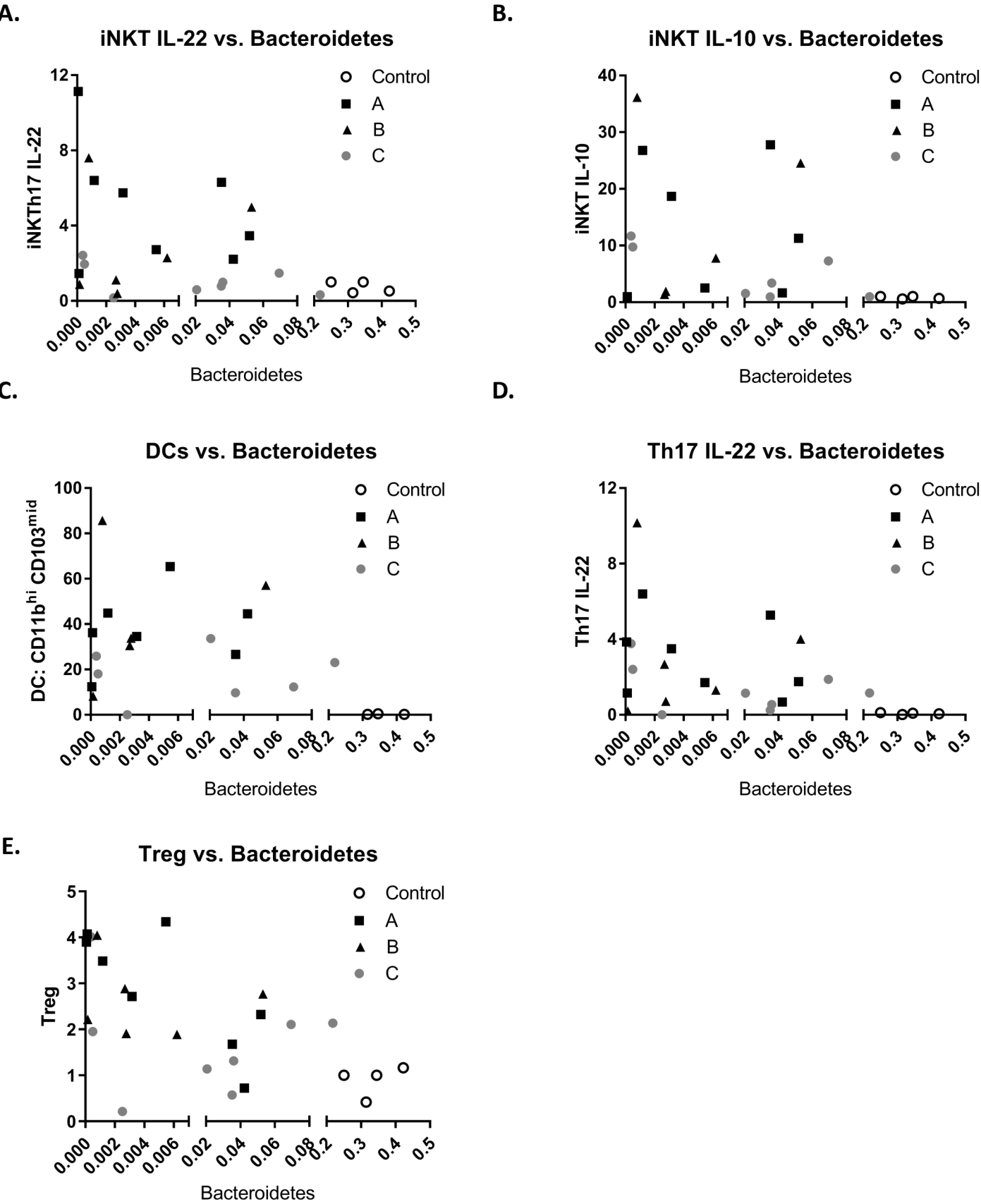

Supplement: Supplementary file 7 — Additional file 7: Figure S6. Immune cell populations correlate to Bacteroidetes with distinct patterns based on surgical segments. Correlation graphs of (A) iNKT (TCRβ+ CD1dtet+) IL-22+ cells, (B) iNKT IL-10+, (C) CD11bhi CD103mid DCs (CD45+ IA-IE+ CD110+), (D) Th17 (TCRβhi CD4+ IL-17A+ IL17F+) and (E) Treg (TCRβ+ CD4+ FoxP3+) versus Bacteroidetes within surgical segments. [file 13099_2021_447_MOESM7_ESM.pdf]

Supp. Figure 7.

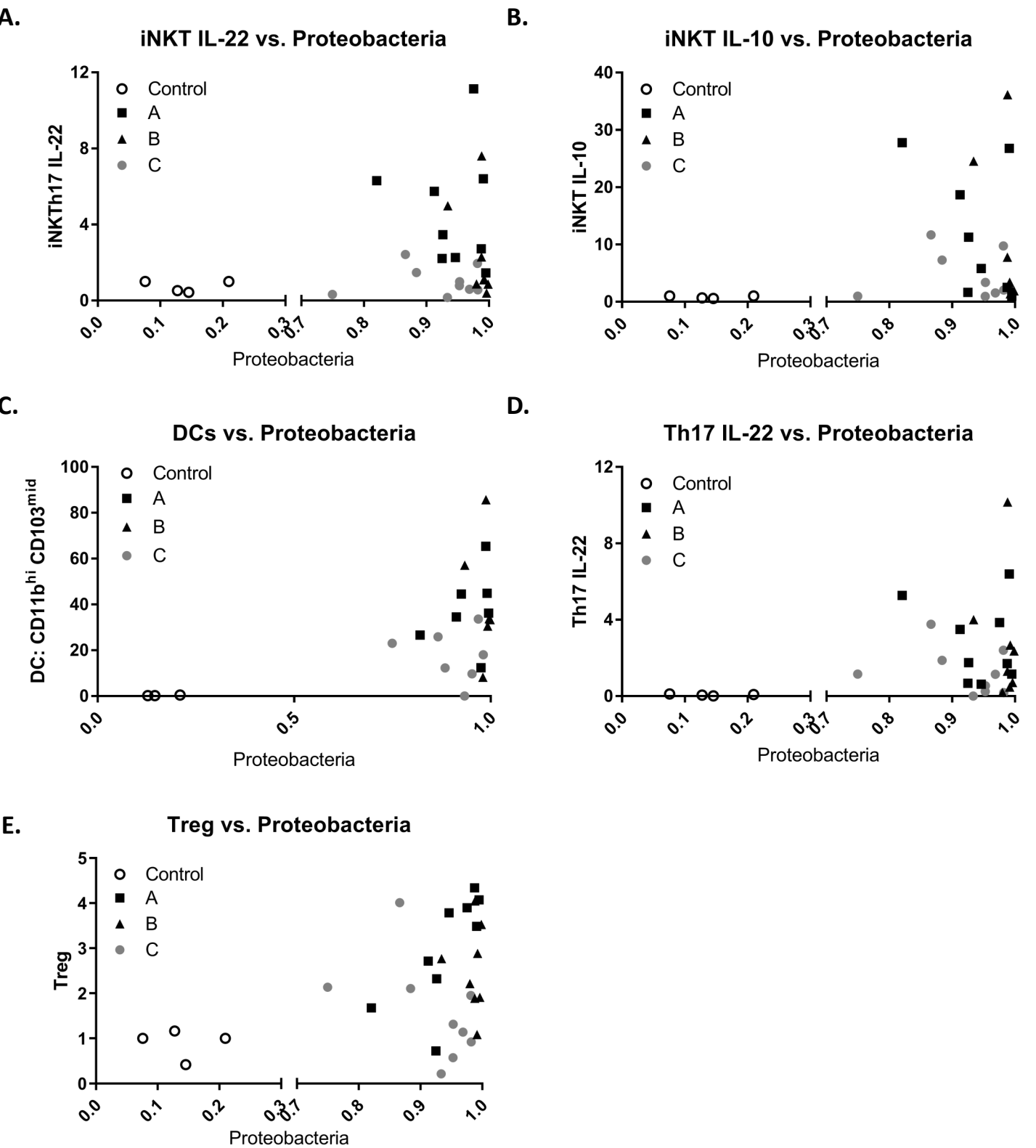

Supplement: Supplementary file 8 — Additional file 8: Figure S7. Immune cell populations correlate to Proteobacteria with distinct patterns based on surgical segments. Correlation graphs of (A) iNKT (TCRβ+ CD1dtet+) IL-22+ cells, (B) iNKT IL-10+, (C) CD11bhi CD103mid DCs (CD45+ IA-IE+ CD110+), (D) Th17 (TCRβhi CD4+ IL-17A+ IL17F+) and (E) Treg (TCRβ+ CD4+ FoxP3+) versus Proteobacteria within surgical segments. [file 13099_2021_447_MOESM8_ESM.pdf]
