# Supplementary material for: Microbiome diversity declines while distinct expansions of Th17, iNKT, and dendritic cell subpopulations emerge after anastomosis surgery
Source: Gut Pathog. 2021 Aug 10;13:51. doi: 10.1186/s13099-021-00447-z (PMC8353768; doi:10.1186/s13099-021-00447-z)
Supplement: Supplementary file 9 — Additional file 9: Table S1. Pearson correlation coefficients (r) for immune cell population versus phylums within anastomoses segments. Bolded r value with * have a p ≤ 0.05 and indicates a significant correlation between the immune cell population and phylum within that surgical segment. [file 13099_2021_447_MOESM9_ESM.pdf]

Supp. Table 1.

Pearson correlation coefficient, r	Control	Segment A	Segment B	Segment C
iNKT IL-22 vs. Bacteroidetes	-0.4894	-0.3118	0.3392	-0.4098
iNKT IL-10 vs. Bacteroidetes	-0.4412	-0.02952	0.3245	-0.4995
DCs vs. Bacteroidetes	-0.3533	0.02185	0.2623	0.1635
Th17 vs. Bacteroidetes	-0.4111	-0.2859	0.07618	-0.15
Treg vs. Bacteroidetes	0.3629	**-0.8083** *	0.9424	0.8040
iNKT IL-22 vs. Firmicutes	0.6123	0.2323	0.3431	0.528
iNKT IL-10 vs. Firmicutes	0.5967	0.5867	**0.8186** *	**0.719** *
DCs vs. Firmicutes	0.6096	-0.4021	-0.02808	-0.006609
Th17 vs. Firmicutes	0.7183	0.3504	0.1259	0.6544
Treg vs. Firmicutes	0.8850	0.2248	0.9777	**0.0428** *
iNKT IL-22 vs. Proteobacteria	0.04811	-0.1008	-0.455	0.03368
iNKT IL-10 vs. Proteobacteria	0.03837	-0.4981	-0.5213	-0.003072
DCs vs. Proteobacteria	0.996	0.3173	-0.2397	-0.1618
Th17 vs. Proteobacteria	-0.2696	-0.2188	-0.1599	-0.2885
Treg vs. Proteobacteria	0.9007	**0.0282** *	0.8947	0.1661
