# Supplementary material for: Microbiome diversity declines while distinct expansions of Th17, iNKT, and dendritic cell subpopulations emerge after anastomosis surgery
Source: Gut Pathog. 2021 Aug 10;13:51. doi: 10.1186/s13099-021-00447-z (PMC8353768; doi:10.1186/s13099-021-00447-z)
Supplement: Supplementary file 10 — Additional file 10: Table 2. Pearson correlation coefficients (r) for immune cell population versus phylums within surgery types. Bolded r value with * have a p ≤ 0.05 and indicates a significant correlation between the immune cell population and phylum within that type of surgery. [file 13099_2021_447_MOESM10_ESM.pdf]

Supp. Table 2.

Pearson correlation coefficient, r	Control	End-to-end Anastomosis	Roux-en-Y
**iNKT IL-22 vs. Bacteroidetes**	-0.4894	-0.4255	-0.1539
**iNKT IL-10 vs. Bacteroidetes**	-0.4412	-0.3247	-0.1628
**DCs vs. Bacteroidetes**	-0.3533	-0.3291	0.07444
**Th17 vs. Bacteroidetes**	-0.4111	-0.2713	-0.198
**Treg vs. Bacteroidetes**	0.3629	-0.3311	-0.2634
**iNKT IL-22 vs. Firmicutes**	0.6123	0.5621	0.1521
**iNKT IL-10 vs. Firmicutes**	0.5967	**0.8138** *	0.2951
**DCs vs. Firmicutes**	0.6096	-0.1984	-0.3143
**Th17 vs. Firmicutes**	0.7183	**0.8151** *	0.073
**Treg vs. Firmicutes**	0.1150	0.2151	-0.1493
**iNKT IL-22 vs. Proteobacteria**	0.04811	0.007948	-0.08512
**iNKT IL-10 vs. Proteobacteria**	0.03837	-0.2088	-0.2126
**DCs vs. Proteobacteria**	0.996	0.3957	0.1931
**Th17 vs. Proteobacteria**	-0.2696	-0.1969	-0.01882
**Treg vs. Proteobacteria**	-0.09934	0.1908	0.2217
